# Adaptation of the Patient Benefit Assessment Scale for Hospitalised Older Patients: development, reliability and validity of the P-BAS picture version

**DOI:** 10.1186/s12877-021-02708-7

**Published:** 2022-01-11

**Authors:** Maria Johanna van der Kluit, Geke J. Dijkstra, Sophia E. de Rooij

**Affiliations:** 1 University of Groningen, University Medical Center Groningen, University Center for Geriatric Medicine, Hanzeplein 1, Groningen, 9700 RB The Netherlands; 2 Department of Health Sciences, Applied Health Research, University of Groningen, University Medical Center Groningen, Groningen, The Netherlands; 3 Research Group Living, Wellbeing and Care for Older People, NHL Stenden University of Applied Sciences, Leeuwarden, The Netherlands

**Keywords:** Older adults, Hospitalisation, Patient perspective, Goal setting, Patient-reported outcomes, Validity, Reliability, Responsiveness, Minimal important change (MIC), Value-based health care

## Abstract

**Background:**

The Patient Benefit Assessment Scale for Hospitalised Older Patients (P-BAS HOP) is a tool developed to both identify the priorities of the individual patient and to measure the outcomes relevant to him/her, resulting in a Patient Benefit Index (PBI), indicating how much benefit the patient had experienced from the hospitalisation. The reliability and the validity of the P-BAS HOP appeared to be not yet satisfactory and therefore the aims of this study were to adapt the P-BAS HOP and transform it into a picture version, resulting in the P-BAS-P, and to evaluate its feasibility, reliability, validity, responsiveness and interpretability.

**Methods:**

Process of instrument development and evaluation performed among hospitalised older patients including pilot tests using Three-Step Test-Interviews (TSTI), test-retest reliability on baseline and follow-up, comparing the PBI with Intraclass Correlation Coefficient (ICC), and hypothesis testing to evaluate the construct validity. Responsiveness of individual P-BAS-P scores and the PBI with two different weighing schemes were evaluated using anchor questions. Interpretability of the PBI was evaluated with the visual anchor-based minimal important change (MIC) distribution method and computation of smallest detectable change (SDC) based on ICC.

**Results:**

Fourteen hospitalised older patients participated in TSTIs at baseline and 13 at follow-up after discharge. After several adaptations, the P-BAS-P appeared feasible with good interviewer’s instructions. The pictures were considered relevant and helpful by the participants. Reliability was tested with 41 participants at baseline and 50 at follow-up. ICC between PBI_1_ and PBI_2_ of baseline test and retest was 0.76, respectively 0.73. At follow-up 0.86, respectively 0.85.

For the construct validity, tested in 169 participants, hypotheses regarding importance of goals were confirmed. Regarding status of goals, only the follow-up status was confirmed, baseline and change were not. The responsiveness of the individual scores and PBI were weak, resulting in poor interpretability with many misclassifications. The SDC was larger than the MIC.

**Conclusions:**

The P-BAS-P appeared to be a feasible instrument, but there were methodological barriers for the evaluation of the reliability, validity, and responsiveness. We therefore recommend further research into the P-BAS-P.

**Supplementary Information:**

The online version contains supplementary material available at 10.1186/s12877-021-02708-7.

## Background

Quantitative outcomes of hospitalisation can be described with objective or subjective measures. Objective measures are, for example, length of stay, mortality, or clinical performance indicators. Subjective measures are patient-reported outcomes and are dependent on the judgment of the individual patient; examples are symptom burden, functional status, and health-related quality of life [1]. These kind of outcomes are often measured to compare different treatments, the effectiveness of alternatives of hospital admission, such as hospital at home [2, 3], acute geriatric community hospital [4], or the effectiveness of better geriatric management of in-hospital patients [5]. However, patient-reported outcomes do not always reflect what patients find important since patient involvement in the development of instruments is rare [6]. But even when patients are involved in the generation of outcomes, they often only reflect the priorities of the overall patient population and not of the individual patient, while which outcomes are considered important, differ per individual [7, 8]. We therefore developed the Patient Benefit Assessment Scale for Hospitalised Older Patients (P-BAS HOP) [9].

### P-BAS HOP

The P-BAS HOP was designed as a tool to inventory the personal goals and benefits of individual older hospitalised patients. The development and validation of the first version was published previously [9, 10]. In brief: The P-BAS HOP is an interview-based questionnaire consisting of two parts: 1) a baseline questionnaire to select and assess the importance of various predefined personal goals derived from qualitative interviews with hospitalised older patients and 2) an evaluation questionnaire to assess the extent to which the hospital admission helped to achieve these individual goals. Based on these data an individual Patient Benefit Index (PBI) is computed, which is an overall value reflecting the achievement of the goals weighted by their importance.

The reliability and the validity of the P-BAS HOP appeared to be not yet satisfactory. We therefore recommended adapting the P-BAS HOP as follows: modify the first step in which the participant was asked whether he experienced a problem or limitation with a subject, differentiate between prevention, preservation and improvement, and remove the word ‘again’ in the questions. Also, reformulate the questions in the follow-up questionnaire or make clear to which timeframe they refer [10].

### Development P-BAS picture version

To further enhance the understanding of the P-BAS HOP, as 29% of the Dutch population have limited health literacy, and this prevalence is higher among older people [11], also a picture version was made [12]. All items were changed into drawings by a professional illustrator. These drawings were presented to older patients visiting or staying in the hospital to check comprehensibility and were modified when necessary. The final pictures were printed on plasticized cards. Initially, the original format was maintained in which first a selection was made of limitations on one side of the card and then a second part whether the applicable cards were a goal and, if so, how important this goal was. But a small pilot revealed that, although the cards were attractive to participants, the limitations found in the original format remained [10]. We, therefore, decided to develop and test an adapted format.

### New format of the P-BAS picture version (P-BAS-P)

Definitions of the most relevant concepts applied in the P-BAS-P are provided in Table 1.

**Table 1:** Definitions of concepts in the P-BAS-P

Concept	Definition
Goal	Personal outcome a participant hopes to achieve with the hospitalisation.
Importance	How important a goal is according to the participant, varying from not at all important to very important.
Status	How is it going with a particular goal according to the participant, varying from very bad to very good.
Prevention	Only used for symptoms. The goal of the participant is to prevent a symptom.
Preservation	Used for all other goals. The goal is to preserve a function/condition.
Improvement	The goal is to improve symptom/condition/functioning.
Change	Difference in status between follow-up and baseline.
Score (S)	Component of the PBI. Combination of change and prevention/preservation or improvement. If the goal was prevention/preservation S = Change, if the goal was improvement S = Change - 1. See Additional file 1, section *Calculation of the PBI* for the full explanation.
Patient Benefit Index (PBI)	Overall value reflecting the achievement of goals weighted by their importance. PBI_1_ is PBI with linear weighting scheme, and PBI_2_ is PBI with quadratic weighting scheme.

#### Baseline during hospitalisation

**Fig. 1:**
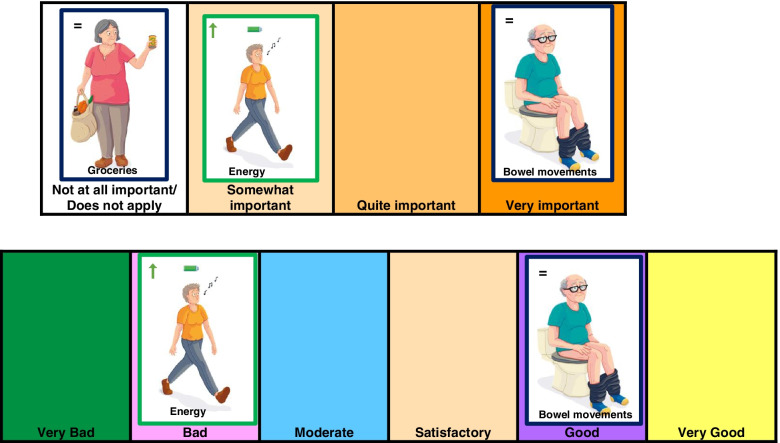
Example of cards and answer sheets of P-BAS-P. Above: answer sheet importance, under: answer sheet status

A set of cards with 21 possible goals, of each goal two identical cards, were presented to the participant. Two sheets with answer options were placed in front of the participant (Fig. 1). The participant was asked per card whether this goal applied to him. When a goal did not apply or was not important for the participant, he/she placed the cards in the box ‘not at all important/does not apply’ of the ‘importance sheet’. When the participant indicated that a goal applied to him/her, the following questions were answered (flexible order): 1) indicate the importance of the goal by placing the card in one of the boxes ‘somewhat’, ‘quite’ or ‘very’ important on the ‘importance sheet’. 2) indicate how it was going on the day of admission with the particular subject by placing the card in one of the boxes ‘very bad’, ‘bad’, ‘mediocre’, ‘satisfactory’, ‘good’, ‘very good’ on the ‘status sheet’. 3) indicate whether the goal is prevention/preservation or improvement, by turning up the blue side with = −sign or the green side with the ↑-sign. The exceptions were ‘knowing what is wrong with me’ and ‘remaining alive’. Since for these two goals the options prevention/preservation or improvement were not applicable, these cards had no = − or ↑-sign and had a yellow colour. In addition, for the goal ‘remaining alive’, only one card was available, since we only asked for the importance and not the status. The last cards depicted a question mark, allowing the participant the opportunity to add an extra personal goal. When all cards were placed, the interviewer wrote the answers on an answer sheet. The complete instructions and answer sheet are shown in Additional file 1.

#### Follow-up

During the follow-up, only goals applicable for that participant were evaluated. Two formats were tested:

- Format one: the participant was asked what the status per item was at that moment with the answer options: ‘very bad’, ‘bad’, ‘mediocre’, ‘satisfactory’, ‘good’, ‘very good’.
- Format two: when the goal was prevention, the follow-up question was formulated as: ‘Because of the hospitalisation…. was prevented’, when the goal was preservation, the follow-up question was formulated as: ‘Because of the hospitalisation, I still…’, when the goal was improvement, the follow-up question was formulated as: ‘Because of the hospitalisation, I can better/have more/am less… ‘. In all cases, the answer options were: ‘not at all’, ‘somewhat’, ‘quite’, ‘completely’.

#### Calculation of the PBI

The calculation of the PBI format one is based on the achievement of the set goals, weighted for their importance. The full calculation is explained in Additional file 1.

## Methods

**Fig. 2:**
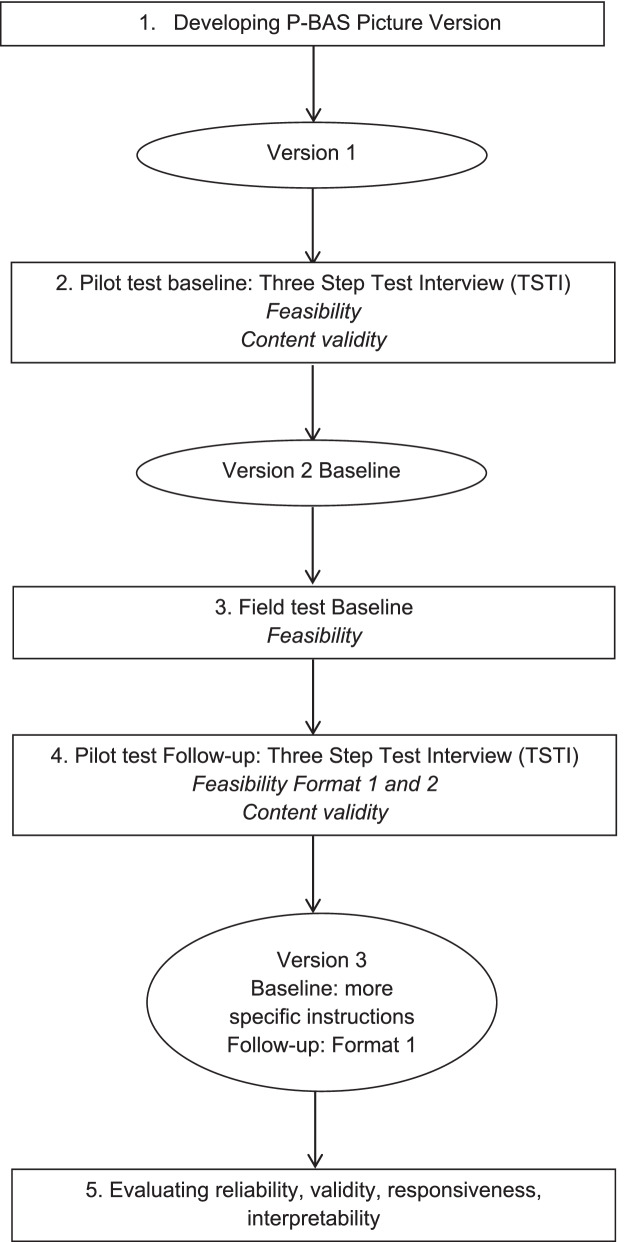
Steps used to develop and test the P-BAS-P

The steps used to develop and test the P-BAS-P are based on the steps of De Vet et al. [13] and outlined in Fig. 2. After each step, the tool was modified. The steps are explained in the following sections. For the readability, the methods and results of each step are alternated.

### Pilot test baseline: three-step test-interview (TSTI)

The first version was tested with the TSTI [14, 15]. The TSTI is a type of cognitive interview useful for assessing how people interpret a questionnaire, its different items, and what types of strategies they use in responding to them. The steps are explained in Additional file 2.

#### Participants

Eligible participants of the TSTI were 70 years and older; planned or unplanned hospitalised on medical or surgical wards of a university teaching hospital in the Netherlands; able to speak and understand Dutch and were without cognitive impairment. Inclusion criteria were verified with the staff nurse, and patients were then approached by the observer (MJvdK). Participants were completely anonymous, no list with names or other identifying data was made, nor did the researchers have access to medical records. Participants gave verbal consent to the interview and audio recording.

#### Data analysis

Data gathering and data analysis were alternated. Interviews were audio recorded and transcribed verbatim. All remarks were then organised by question and step. After that, the data were coded by MJvdK and grouped into categories. The tool was adapted several times after the feedback until it was considered feasible and understandable.

#### Results

Twenty-five older hospitalised patients were approached for the TSTI of the baseline test and fourteen (56%) agreed to participate. Characteristics of the participants are displayed in the second column of Table 2.

**Table 2:** Participants three-steps test-interview (TSTI) baseline and follow-up

Characteristic	Baseline (*n* = 14)	Follow-up (*n* = 13)
	n	n
Gender		
Male	12	8
Female	2	5
Age (years)		
70-79	9	9
80-89	4	4
90-99	1	0
Educational level^a^		
Low	8	3
Middle	2	5
High	3	5
Unknown	1	0
Admission reason^b^		
Pulmonary problems	5	0
Cardiac problems	4	8
Bowel problems	2	3
Kidney problems	2	0
Stroke	0	1
General malaise	1	1

^a^Educational level: Low = no education, primary school, basic vocational training; Middle = secondary education, vocational training; High = bachelor, master

^b^Reason according to the patient

##### Feasibility

Comprehension of the format varied widely between participants. Some participants needed only a short introduction and an example and were then able to place the cards independently in the boxes with only a little guidance from the interviewer, whereas others needed constant guiding and reminding of the aim. Some of these participants had the tendency to elaborate on how they were coping with the subject on the card without specifying whether it was a goal or not. This emphasized the necessity for adequate interviewer instructions and an instruction guide for the interviewers.

An example of guiding by the interviewer:

Washing and dressing. Is that a goal for you with this hospitalisation?

*Yes, that you can still do that yourself.*

Yes. And, uh.

*I find that somewhat important.*

Somewhat important. Because how is it going with washing and dressing now?

*It is still fine, but with difficulty.*

Yes. Satisfactory or good?

*Let’s say satisfactory.*

And is it your goal to preserve it that way or to improve it?

*To preserve it that way, yes.* (P1)

Example of a participant with the tendency to elaborate on how he was coping with the subject, without specifying whether it was a goal:

*Well, the next one depicts a figure with a lot of pain, I can say that I have a lot of pain, but I have, I don't feel pain that easily. But when I move, I do feel pain now. And you have to indicate that here in points, I have always found that very difficult to interpret, but eh, I, I say, I, I, when I lay down it is not too bad and when I move, it hurts, I'm in the midst of it. So I have pain.*

Yes. Yes. And is it a goal for you to reduce this?

*Yes! Otherwise I will die. (laughing) (P7)*

##### Adaptations

The following adaptations were made to the format:

Originally we had four answer options: ‘bad, mediocre, satisfactory, good’, but this appeared insufficient for some participants. We therefore added ‘very bad’ and ‘very good’.

Initially, we had one card for each goal and one answering sheet with a horizontal axis for status and a vertical axis for importance where the participant could place the card. Since this appeared complicated for participants, we made two different answer sheets: one for status, and one for importance. The participant had two cards per goal and placed one card on each sheet, as explained in the methods.

Since most interviews were conducted after a few days of hospitalisation, the question about status was sometimes problematic. As some participants indicated that complaints had already improved compared to the day they were admitted, they had the goal ‘improving’, but their status was ‘good’ or ‘very good’ at the moment of interview. We therefore decided to ask in these cases how the status was on the day of admission.

We changed the text on the card ‘visiting’. Originally we had the text ‘visiting family or friends’, but some participants started elaborating about family and friends that had passed away. To help them focus only on visiting, we changed the text into ‘visiting’.

A user guide was written for the interviewers.

##### Opinions of participants

A broad variety of evaluations by participants included: pleasant diversion; nice pictures; emotional to be confronted with own impairments; subjects were considered very relevant; it was helpful to express wishes and concerns; interesting; and somewhat tiring.

##### Content validity

The goals the participants mentioned in their own words were indicated in the tool as at least ‘somewhat important’ in all cases.

### Field test baseline

Version 2 was tested with a new group of hospitalised older patients. The aim of this field test was to train the research assistants in the use of the P-BAS-P and understand its feasibility.

#### Participants

Eligible participants were consecutive patients aged 70 years and older; planned or unplanned hospitalised on medical or surgical wards of a university teaching hospital, expected to stay for at least 48 h; and a maximum of 4 days in hospital at the moment of interviewing; able to speak and understand Dutch and were without cognitive impairment. Inclusion criteria were verified with the staff nurse. Patients were approached by a trained research assistant and gave signed informed consent to participate.

#### Results

Research assistants practised with Version 2 of the P-BAS-P in a group of 62 consecutive hospitalised older patients. Sample characteristics are shown in the second column of Table 3. Through observation and feedback it was revealed that the instruction to ask for the status on the day of admission when the interview was conducted after a few days and the status of the participant had changed, was not clear for all research assistants. We therefore decided to change the instructions and always ask for the status on the day of admission.

**Table 3:** Sample characteristics field test baseline and evaluation reliability, validity, responsiveness, interpretability

Characteristic	Field test baseline (*n* = 62)	Evaluation reliability, validity, responsiveness, interpretability (*n* = 169)
	n (%)	n (%)
Gender		
Male	37 (60)	96 (57)
Female	25 (40)	73 (43)
Age (years), median (range)	74 (70–96)	75 (70–98)
Living situation		
Independent	61 (98)	162 (96)
Sheltered accommodation	0	4 (2)
Senior home	1 (2)	2 (1)
Nursing home	0	1 (1)
Educational level^a^		
Low	10 (16)	61 (36)
Middle	37 (60)	65 (38)
High	15 (24)	43 (25)
Specialty		
Medical	19 (31)	78 (46)
Surgical	21 (34)	36 (21)
Intervention cardiology	18 (29)	48 (28)
Unknown	4 (6)	7 (4)
Admission type		
Acute	32 (52)	75 (44)
Elective	26 (45)	87 (52)
Unknown	4 (6)	7 (4)
Length of stay (days) median (range)	4 (1–28)	5 (1–35)
Number of days the interview took place after admission		
1	1 (2)	10 (6)
2	28 (45)	64 (38)
3	24 (40)	69 (41)
4	8 (13)	26 (15)

^a^Educational level: Low = no education, primary school, prevocational education; Middle = secondary or vocational education; High = bachelor, master

### Pilot test follow-up: three-step test-interview (TSTI)

The TSTI was repeated with the follow-up questionnaire 4 to 10 weeks after discharge at the home of the participant. As described in paragraph ‘New Format of the P-BAS-P’, two formats were tested. Format one: the participant was asked what the status per item was at that moment. Format two started with ‘Because of the hospitalisation…’ and the rest of the sentence depended on the goal being ‘prevention’, ‘preservation’, or ‘improvement’.

The follow-up questionnaire was filled out in writing by the participant, in the presence of one observer/interviewer. Other steps and the analysis of the TSTI were conducted in the same manner as in step 2.

#### Participants

Participants included in the Field test Baseline (step 3) were approached during hospitalisation, received written information, and were asked permission to be approached after hospital discharge. From 4 weeks after discharge, participants were called to provide information again and to make an appointment if appropriate. Informed consent was signed at the home visit before the TSTI.

#### Results

Eighteen participants were approached in the hospital, and 17 gave permission to be contacted after discharge. Afterward, two participants refused, one participant appeared too confused for informed consent, and for one participant the opportunity to make an appointment fell outside the timeframe; this resulted in thirteen (72%) participants for the TSTI of the follow-up. Characteristics of participants are displayed in the last column of Table 2.

##### Feasibility format one

Format one was well understood, and answers were given easily. The only confusion was caused by the question ‘How is your disease or condition now?’; as many participants have multiple, it was not clear for all which one was meant. We therefore made the addition: ‘as concerning the disease or condition you were admitted for’, which was well-understood.

##### Feasibility format two

Format two was more problematic. Participants often had to read the sentence several times before it was understood. Some other problems:

Originally the follow-up question in the case of preservation was formulated as: ‘Because of the hospitalisation, I still…’ This caused confusion when the participant already had a low level of baseline function. We therefore changed the sentence into ‘Because of the hospitalisation I maintained…’. Although this gave sometimes complicated sentences, it was better understood.

The question ‘Because of the hospitalisation I remained alive’ was often considered complicated. For example:

*Yes, so I don’t know, if I had surgery or not, if I would not have had surgery, whether I then, whether that was life-threatening. I don’t know. (P8)*

The use of the answer option ‘somewhat’ was interesting, since it was used with many intentions. Often it meant ‘I don’t know’, or ‘actually it is going really badly, but I stay hopeful’, or ‘I don’t want to be too negative’. Only in a minority of the cases it was used to indicate ‘I accomplished my goal to some extent’.

The main concern with format two was that it was unclear which timeframes the participant had to compare. Sometimes participants were evaluating the period in hospital, sometimes shortly after discharge. But even when participants evaluated their current situation, it was not clear with which period they had to compare, this could be long before hospitalisation, shortly before hospitalisation, or during hospitalisation. Changing the words ‘because of’ into ‘thanks to, did not help.

Example:

*Thanks to hospitalisation I have more energy. Well, no more energy. When I compare that with the situation before that time. Well, well, not more. Well, somewhat maybe. Yes… It depends on what I'm comparing it to, by the way. If it was right before my hospital admission, then of course it is eh… then it is completely. But if I go a little further back in time, it is not even that big of a difference. So I find it a little bit difficult to answer, this question. Let me answer it as quite. But more because I don't know at what point in time I have to compare it to. (P13)*

Because of the difficulties with format two, we decided to continue only with format one.

### Evaluating reliability, validity, responsiveness, interpretability

Version 3 was the final version and consisted of the baseline version with changed instructions and follow-up version format one.

This longitudinal study was performed among a new group of hospitalised older patients. The inclusion criteria were identical to step 3. The first face-to-face interview took place in the first 4 days of hospitalisation. The follow-up interview was performed 3 months after discharge by telephone.

#### Reliability

Test-retest reliability of the baseline questionnaire was performed in a smaller group with an interval of 1 day, while the participant was still hospitalised. The participant was not notified in advance of the retest, but was asked for permission for another test on the other day.

Test-retest of the follow-up questionnaire was performed in another sample than the baseline test-retest with an interval of 5 to 10 days. At the end of the first follow-up interview, participants were asked permission to be called back a week later to repeat some questions, without specifying which questions (only P-BAS-P). After the retest, the patient was asked whether anything had changed since the last test.

The PBI was calculated for both test and retest of baseline and follow-up and compared with 2-way random Intraclass Correlation Coefficient (ICC) for absolute agreement [13, 16]. An ICC of at least 0.70 was considered reliable [13, 17].

#### Validity

For the construct validity, the questionnaires or constructs used are summarised in Table 4. Full details are given in Additional file 3.

**Table 4:** Constructs measured for the construct validity

Construct	Operationalisation
Appetite	Dutch VMS screening program (VMS) [18]
Symptoms experienced on admission day	Rotterdam Symptom Checklist (RSCL) [19]
Pain, experienced at moment of interview	Numeric rating scale (NRS) pain (0: no pain at all to 10: the worst imaginable pain)
Fatigue, experienced at moment of interview	NRS fatigue (0: no fatigue at all to 10: the worst imaginable fatigue)
Health-related quality of life at moment of interview	EQ-5D [20]
Admission reason	Acute/ elective; diagnostic/ curative/ palliative
Physical activity	Single question: ‘How often are you physically active for at least 30 min?’ with Likert scale
Social functioning	36-Item Short Form Survey Instrument (SF-36) – Social functioning [21]
Goals on hospital admission	Open question: ‘What do you hope to accomplish with this hospitalisation?’

##### Hypotheses importance of goals

To test the construct validity of the importance of goals, we hypothesised that participants experiencing certain symptoms or limitations score higher on the importance of improving the related goal and that goals mentioned by participants after the open question were indicated as at least ‘somewhat important’ for the concerning goal. The specific goals we developed are listed in Table 5.

**Table 5:** Hypotheses importance of goals

Hypothesis		Expected correlation	Calculated correlation		C/R^a^
			n		
1	Participants who indicated a lack of appetite on the VMS and/or the RSCL are expected to assign higher importance to the goal ‘improving appetite’.	Cramér’s V > 0.10	169	0.41	C
2	Participants who indicated tiredness and/or lack of energy on the RSCL are expected to assign higher importance to the goal ‘improving energy’.	Cramér’s V > 0.10	169	0.26	C
3	Participants who indicated diarrhoea and/or constipation on the RSCL are expected to assign higher importance to the goal ‘improving bowel movements’.	Cramér’s V > 0.10	169	n.c.	n.a.
4	Participants who indicated shortness of breath on the RSCL are expected to assign higher importance to the goal ‘reducing shortness of breath’.	Cramér’s V > 0.10	169	0.68	C
5	Participants who indicated some problems or confined to bed on the EQ-5D mobility, are expected to assign higher importance to the goal ‘improving walking’.	Cramér’s V > 0.10	169	0.41	C
6	Participants who indicated some problems or unable on the EQ-5D self-care, are expected to assign higher importance to the goal ‘improving washing/dressing’.	Cramér’s V > 0.10	169	0.37	C
7	Participants who indicated some problems or unable on the EQ-5D usual activities, are expected to assign higher importance for to goal ‘improving hobbies’.	Cramér’s V > 0.10	166	0.37	C
8	Participants who had an acute admission and/or a diagnostic admission reason, are expected to assign higher importance to the goal ‘knowing what is wrong with me’.	Cramér’s V > 0.10	161	0.20	C
9	Participants with a higher NRS Pain, are expected to assign higher importance to the goal ‘reducing pain’.	Spearman’s > 0.10	164	0.34	C
10	Participants with a higher score on the SF-36 – Social functioning, are expected to assign higher importance to the goal ‘improving visiting’.	Spearman’s > 0.10	164	0.19	C
11	Participants with a lower score on the EQ-5D thermometer ‘general health’, are expected to assign higher importance to the goal ‘feeling better’.	Spearman’s < −0.10	168	− 0.05	R
12	Goals that were mentioned after the open question, are, when applicable, indicated as minimum ‘somewhat important’ for the concerning goal.	Percentage of agreement ≥75%	50	89%	C

^a^*C* = Confirmed, *R =* Rejected

##### Analysis

Hypotheses 1 to 8 were evaluated using Cramér’s V statistic. Hypotheses 9 to 11 with Spearman’s rank-order correlation. Since experiencing a symptom or restraint in a certain subject does not necessarily mean that this is an (important) goal for hospital admission, hypotheses are confirmed if the correlation exceeds ‘small’ as defined by Cohen [22], meaning the correlation > 0.10. Because the assumptions of Cramér’s V statistic were not met because of too low (expected) cell prevalence, for these analyses, the categories ‘somewhat’ and ‘quite important’ were combined.

For hypothesis 12, a random selection of 50 cases was made and goals mentioned in the open question were coded using the item names of the P-BAS-P. When a participant mentioned a goal that was not in the P-BAS-P, it was coded as ‘other’. The coding was done by two researchers independently and then compared and discrepancies were solved by consensus. Subsequently, the percentage of agreement between the codes and answers given in the P-BAS-P was calculated.

The importance of goals was considered valid if at least 75%, thus nine of the first 11 hypotheses were confirmed and hypothesis 12 was confirmed in at least 75% of selected cases [17].

##### Hypotheses status

To test the construct validity of the status of the P-BAS-P, we compared the status of the goals at baseline, follow-up, and change with other validated scales. The specific hypotheses we developed are listed in Table 6.

**Table 6:** Hypotheses status baseline, follow-up, change

		Baseline				Follow-up				Change			
		Expected correlation	n	Calculated correlation	C/R^a^	Expected correlation	n	Calculated correlation	C/R^a^	Expected correlation	n	Calculated correlation	C/R^a^
1	There is a negative correlation between EQ-5D mobility and status for the goal ‘walking’.	rs < − 0.20	108	rs = − 0.27	C	rs < − 0.40	88	rs = − 0.52	C	rs < − 0.30	84	rs = − 0.24	R
2	There is a negative correlation between EQ-5D self-care and status for the goal ‘washing/dressing’.	rs < − 0.20	61	rs = − 0.52	C	rs < − 0.40	55	rs = − 0.74	C	rs < − 0.30	47	rs = − 0.42	C
3	There is a negative correlation between EQ-5D usual activities and status for the goal ‘hobbies’.	rs < − 0.20	77	rs = − 0.33	C	rs < − 0.40	62	rs = − 0.33	R	rs < − 0.30	55	rs = − 0.31	C
4	There is a negative correlation between EQ-5D pain/discomfort and status for the goal ‘pain’.	rs < − 0.20	65	rs = − 0.15	R	rs < − 0.40	56	rs = − 0.44	C	rs < − 0.30	54	rs = − 0.12	R
5	There is a positive correlation between EQ-5D thermometer ‘general health’ and status for the goal ‘better’.	rs > 0.20	148	rs = 0.19	R	rs > 0.40	130	rs = 0.62	C	rs > 0.30	127	rs = 0.25	R
6	There is a negative correlation between the SF-36 – Social functioning and status for the goal ‘visiting’.	rs < − 0.20	74	rs = − 0.35	C	rs < − 0.40	45	rs = − 0.55	C	rs < − 0.30	41	rs = − 0.16	R
7	There is a positive correlation between physical activity and status for the goal ‘sports’.	rs > 0.20	68	rs = 0.15	R	rs > 0.40	45	rs = 0.20	R	rs > 0.30	40	rs = − 0.24	R
8	There is a negative correlation between NRS Fatigue and status for the goal ‘energy’.	rs < − 0.20	137	rs = − 0.10	R	rs < − 0.40	116	rs = − 0.60	C	rs < − 0.30	109	rs = − 0.22	R

^a^*C =* Confirmed, *R* = Rejected

##### Analysis

Hypotheses were evaluated using Spearman’s rank-order correlation. The P-BAS-P status and other constructs at follow-up both refer to the moment of the interview, while at baseline the P-BAS-P asks for the status on the day of admission and the other constructs for the moment of the interview, which is usually a few days later. Since the participant’s situation can change a lot in that few days, we expected a lower correlation at baseline, and therefore the hypotheses were confirmed at baseline when the correlation > 0.20 and on follow-up > 0.40. For the change scores between follow-up and baseline, hypotheses were confirmed when the correlation > 0.30. The status was considered valid if at least 75%, thus six of the eight hypotheses of baseline, follow-up, and change, respectively, were confirmed [17].

#### Responsiveness of the score and PBI

The following hypothesis was used to test the validity of the scores: Accomplishing goals noted on the open question correlate with the score on the P-BAS-P, if applicable. For this hypothesis, the same records were used as for hypothesis 12 regarding the importance of goals. For dyads with agreement between the code for the open question and the P-BAS-P item, the Spearman’s rank-order correlation between the answer on the open question and the corresponding P-BAS-P score was calculated. A hypothesis was confirmed if the correlation ≥0.50.

The following anchor question was used to validate the PBI: ‘How much have you benefited from the admission?’ With the following answer options: ‘not at all’, ‘a little bit’, ‘somewhat’, ‘much, ‘very much’. We also asked the participants for clarification of their answer.

The PBI is considered valid when it has a Spearman’s correlation coefficient ≥ 0.50 with the anchor question [23, 24]. As the conclusion of how much benefit the participant had was not always based on achieved goals but could also be based on other indicators, for example, how kind the hospital staff was [10], we also calculated the Spearman’s correlation coefficient for the selection of participants who based their anchor only on outcomes. We therefore coded explanations participants gave in ‘based on outcomes’, or ‘based on other grounds’.

#### Interpretability

Interpretability is evaluated with the visual anchor-based minimal important change distribution method [13, 24], with the same anchor question as mentioned above. Participants who indicated: ‘not at all’, and ‘a little bit’, were considered as having no important benefit. Participants who indicated ‘much’ or ‘very much’, are considered as having important benefit. As it was not clear whether ‘somewhat’ was considered as important benefit or not, we labelled this as ‘borderline’. Receiver operating characteristic (ROC) curves were used to determine optimal cut-off points for important and no important benefit.

To assess whether ROC cut-offs lay outside the measurement error, the smallest detectable change (SDC) was computed [13, 25]. The following formula was used: $SDC= SEM\times 1,96\times \sqrt{2}$. Where SEM = Standard Error of Measurement, calculated as: $SEM= SD\sqrt{\left(1- ICC\right)}$, where SD is the pooled standard deviation [13, 16, 25]. As we calculated reliability both at baseline and follow-up, but with different participants, we used the largest SEM for calculating SDC.

#### Missing values

When the P-BAS-P was not administered, this case was completely deleted. For all other missing values, we used pairwise deletion. For calculating the PBI only complete items were used, when only the importance was missing, the importance of these items was set on ‘quite important’.

#### Results

##### Sample

**Fig. 3:**
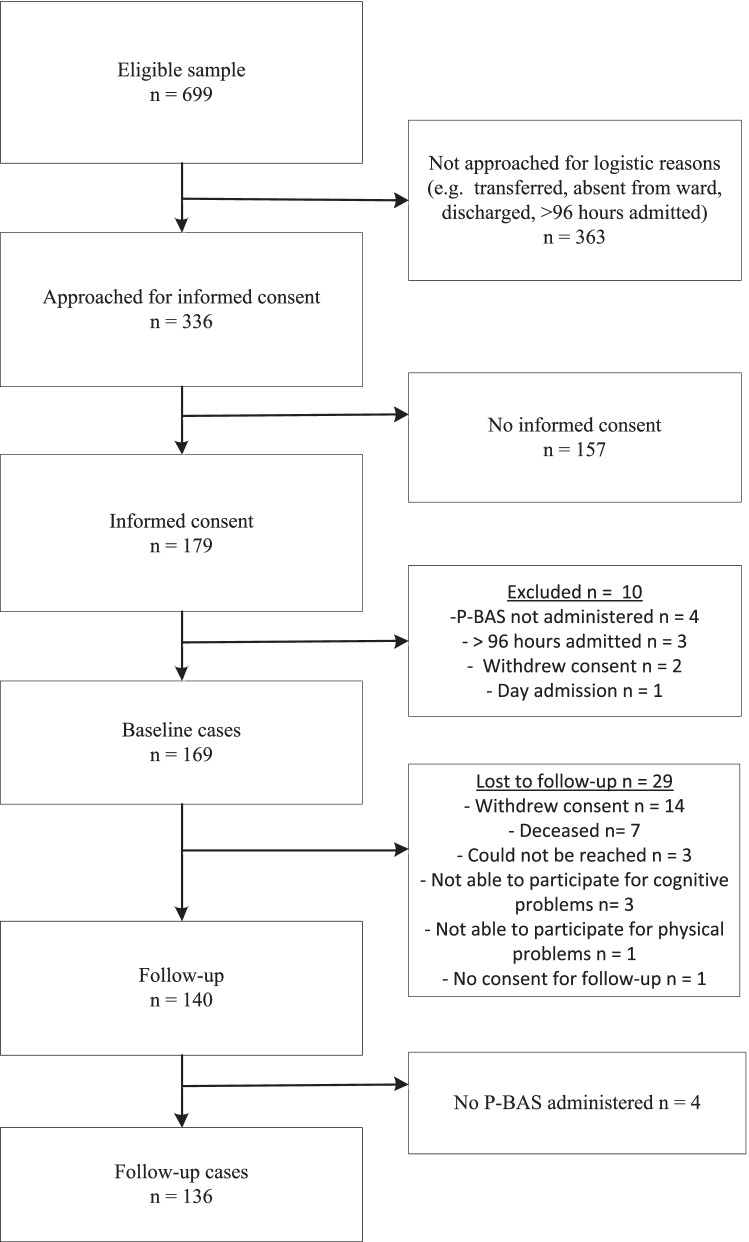
Flowchart participant inclusion for Evaluating Reliability, Validity, Responsiveness, Interpretability

From the 699 eligible patients, 336 were approached for informed consent and 179 gave it. After exclusion of ten cases, we had 169 baseline cases. We lost 29 to follow-up and in an additional four the P-BAS-P were not administered at follow-up, which resulted in 136 follow-up cases. Full details are shown in Fig. 3. Most (41%) baseline interviews were done on the third day after admission.

Sample characteristics are shown in the last column of Table 3, and Additional file 4 shows the scores of the other questionnaires applied for evaluating construct validity.

##### Descriptive statistics P-BAS-P

In two cases the pictures were not used. One case was because there was no table available, and the other because the participant had to lie down completely flat. In both of these cases, the interviewer asked them all questions without using the cards. The time to conduct the P-BAS-P varied from six to 21 min, with a mean of 11 min.

Table 7 shows the baseline descriptive statistics of the P-BAS-P. The number of goals selected as a minimum of ‘somewhat important’ varied from 3 to 21 per person, with a median of 12 and a mean of 11.7. Twenty-eight participants mentioned an extra goal. Examples were: going on holiday, resuming (volunteer) work, or shopping. The missing values at baseline are all due to the interviewer accidentally forgetting to indicate the option on the answer sheet after the interview, except for one participant who did not know to indicate how important the goal ‘enjoying life’ was to him.

**Table 7:** P-BAS-P Baseline descriptive statistics *n* = 169

	Importance					Prevention/preservation/improvement		Status						
	Not at all/ not applicable n (%)	Somewhat n (%)	Quite n (%)	Very n (%)	Missing	Improvement n (%)	Missing	Very bad	Bad	Mediocre	Satisfactory	Good	Very good	Missing
Better	9 (5)	4 (2)	39 (23)	117 (69)	0	148 (88)	0	15 (9)	49 (31)	58 (37)	17 (11)	18 (11)	2 (1)	1
Energy	29 (17)	11 (7)	52 (31)	77 (46)	0	121 (86)	0	19 (14)	44 (31)	47 (34)	18 (13)	8 (6)	4 (3)	0
Pain	100 (59)	7 (4)	19 (11)	43 (35)	0	59 (86)	0	12 (17)	26 (38)	20 (29)	2 (3)	6 (9)	3 (4)	0
Bowel movements	141 (83)	5 (3)	11 (7)	12 (7)	0	19 (68)	0	1 (4)	11 (39)	5 (18)	1 (4)	8 (5)	2 (1)	0
Shortness of breath	71 (42)	3 (2)	27 (16)	68 (40)	0	91 (93)	0	12 (13)	42 (43)	30 (31)	4 (4)	9 (9)	1 (1)	0
Walking	60 (36)	5 (3)	37 (22)	66 (39)	1	93 (85)	0	14 (13)	45 (42)	28 (26)	9 (8)	8 (7)	4 (4)	1
Appetite	126 (75)	5 (3)	24 (14)	14 (8)	0	27 (63)	0	3 (7)	9 (21)	14 (33)	4 (9)	12 (28)	1 (2)	0
Knowing what is wrong	111 (66)	2 (1)	14 (8)	41 (24)	1	n.a.	n.a.	8 (14)	11 (19)	18 (32)	8 (14)	9 (16)	3 (5)	1
Curing	10 (6)	8 (5)	39 (23)	111 (66)	1	123 (79)	4	15 (10)	46 (29)	69 (44)	15 (10)	10 (6)	3 (2)	1
Alive	21 (12)	12 (7)	21 (12)	115 (68)	0	n.a.	n.a.	n.a.	n.a.	n.a.	n.a.	n.a.	n.a.	n.a.
Enjoy	52 (31)	11 (7)	41 (24)	64 (38)	1^a^	76 (65)	0	6 (5)	20 (17)	33 (28)	23 (20)	24 (21)	10 (9)	1
Groceries	81 (48)	11 (7)	44 (26)	33 (20)	0	53 (60)	0	11 (13)	20 (23)	25 (28)	4 (5)	23 (26)	5 (6)	0
Wash and dress	108 (64)	2 (1)	28 (17)	31 (18)	0	27 (44)	0	4 (7)	11 (18)	11 (18)	7 (12)	21 (34)	7 (12)	0
Garden	109 (65)	11 (7)	23 (14)	26 (15)	0	46 (77)	0	3 (5)	21 (35)	13 (22)	8 (13)	14 (23)	1 (2)	0
Sports	98 (58)	13 (8)	29 (17)	29 (17)	0	54 (76)	0	14 (20)	21 (30)	17 (24)	5 (7)	9 (13)	5 (7)	0
Hobbies	91 (54)	11 (7)	23 (14)	44 (26)	0	45 (58)	0	7 (9)	21 (16)	27 (35)	8 (10)	19 (25)	4 (5)	1
Driving	89 (53)	2 (1)	29 (17)	49 (29)	0	25 (31)	0	9 (11)	7 (9)	4 (5)	11 (14)	41 (51)	8 (10)	0
Outings	87 (51)	16 (9)	30 (18)	36 (21)	0	57 (70)	0	9 (11)	26 (32)	19 (23)	4 (5)	20 (24)	4 (5)	0
Visiting	92 (54)	12 (7)	34 (20)	31 (18)	0	37 (48)	0	10 (13)	11 (14)	13 (17)	10 (13)	29 (38)	4 (5)	0
Home	63 (37)	1 (1)	8 (5)	96 (57)	1	11 (10)	0	4 (4)	3 (3)	5 (5)	6 (6)	63 (59)	25 (24)	0
Independence	52 (31)	2 (1)	25 (15)	89 (53)	1	43 (37)	0	10 (9)	18 (15)	15 (13)	14 (12)	45 (39)	15 (13)	0
Extra	141 (83)	1 (1)	9 (5)	18 (11)	0	22 (79)	0	2 (7)	10 (36)	5 (18)	4 (14)	3 (11)	4 (14)	0

^a^Missing values at baseline are all due to the interviewer accidentally forgetting to indicate the option on the answer sheet after the interview, except for ‘enjoying life’, which was unknown by the participant

Table 8 shows the follow-up descriptive statistics and change scores of the P-BAS-P. Since the end of March 2020, the Corona-pandemic influenced answers on the items groceries, sports, hobbies, outings, visiting, and ‘extra’. In cases where participants could not answer this question or indicated the answer was influenced by the Corona-measures, the answer was replaced by ‘missing due to Corona’. A closer look at the other missing values revealed some patterns: In four cases the participant did not know the answer, for example the bowel movements were too irregular, or the participant was still under treatment and did not know to indicate what the disease status was. There were some seasonal problems: at the follow-up moment, it was not the right season for gardening (1x) or the hobby ‘fishing’ (3x). More difficult to interpret were participants stating they did not do an activity, for example: ‘my husband does the groceries’ (1x), ‘I don’t work in the garden’ (3x), ‘I don’t sport’ (4x), ‘we don’t go on outings’ (2x), ‘I have no hobbies anymore’ (1x), ‘I am not allowed to drive’ (1x). It is unsure whether this meant the participant did not reach the goal, so the answer should be ‘very bad’, since the participant mentioned, for example, it was important to garden, and now he does not garden, or whether there was another reason not to garden. In three cases it is doubtful whether the goal selected on baseline was appropriate since the participant stated at follow-up ‘I have never sported/made outings/visited’. One extra goal had a missing value since the goal was ‘becoming 100’, while the participant was 73 years on baseline.

**Table 8:** P-BAS-P Follow-up and change *n* = 136

	n	Very bad	Bad	Mediocre	Satisfactory	Good	Very good	Missing	Missing Corona	Change (F-B)	Score
Better	130	0	3 (2)	31 (24)	30 (23)	58 (43)	8 (6)	0		Mean: 1.35 SD: 1.54 Range: − 2 – 5	Mean: 0.44 SD: 1.48 Range: − 3 - 4
Energy	116	1(1)	7 (6)	40 (35)	25 (22)	40 (35)	3 (3)	0		Mean: 1.15 SD: 1.44 Range: −2 – 4	Mean: 0.28 SD: 1.36 Range: −3 - 3
Pain	57	1 (2)	0	15 (27)	8 (14)	24 (45)	7 (13)	1		Mean: 1.76 SD: 1.65 Range: −2 – 5	Mean: 0.93 SD: 1.49 Range: − 3 - 4
Bowel movements	28	1 (4)	1 (4)	2 (7)	6 (22)	14 (52)	3 (11)	1		Mean: 0.90 SD: 1.67 Range: −1 – 4	Mean: 0.24 SD: 1.48 Range: −2 - 3
Shortness of breath	78	0	4 (5)	24 (31)	13 (17)	32 (41)	5 (6)	0		Mean: 1.52 SD: 1.46 Range: −2 – 4	Mean: 0.59 SD: 1.42 Range: −3 - 3
Walking	89	2 (2)	5 (6)	27 (31)	21 (24)	30 (34)	3 (3)	1		Mean: 1.19 SD: 1.57 Range: − 3 – 4	Mean: 0.33 SD: 1.49 Range: −4 - 3
Appetite	40	1 (3)	2 (5)	5 (13)	5 (13)	21 (53)	6 (15)	0		Mean: 1.00 SD: 1.41 Range: −1 - 4	Mean: 0.38 SD: 1.23 Range: −2 - 3
Knowing what is wrong	58	1 (2)	4 (7)	2 (4)	12 (21)	26 (46)	12 (21)	1		Mean: 1.32 SD: 1.74 Range: −2 – 5	Mean: 0.32 SD: 1.74 Range: −3 - 4
Curing	132	0	6 (5)	26 (20)	25 (19)	53 (41)	19 (15)	3		Mean: 1.63 SD: 1.46 Range: −2 – 5	Mean: 0.84 SD: 1.49 Range: −3 - 5
Alive	117	n.a.	n.a.	n.a.	n.a.	n.a.	n.a.	n.a.	n.a.	n.a.	Mean: 0 SD: 0 Range: 0
Enjoy	105	1 (1)	1 (1)	15 (14)	19 (18)	47 (45)	21 (20)	1		Mean: 1.05 SD: 1.68 Range: −4 - 5	Mean: 0.39 SD: 1.54 Range: − 4 - 4
Groceries	74	4 (6)	6 (9)	9 (13)	8 (11)	38 (54)	6 (9)	1	3	Mean: 0.89 SD: 1.67 Range: −4 - 5	Mean: 0.29 SD: 1.50 Range: −5 - 4
Wash and dress	55	0	4 (7)	5 (9)	5 (9)	32 (58)	9 (16)	0		Mean: 0.85 SD: 1.46 Range: −2 - 5	Mean: 0.36 SD: 1.34 Range: − 2 - 5
Garden	54	3 (6)	7 (14)	8 (16)	10 (7)	17 (34)	5 (10)	4		Mean: 0.85 SD: 1.67 Range: −3 – 5	Mean: 0.06 SD: 1.51 Range: − 3 - 4
Sports	62	3 (7)	8 (17)	8 (17)	8 (17)	14 (30)	5 (11)	4	12	Mean: 0.78 SD: 1.93 Range: −4 – 4	Mean: 0.05 SD: 1.73 Range: − 4 - 3
Hobbies	72	2 (3)	2 (3)	11 (18)	8 (13)	30 (48)	9 (15)	5	5	Mean: 0.64 SD: 1.68 Range: −2 – 5	Mean: 0.45 SD: 1.55 Range: − 3 - 4
Driving	74	5 (7)	4 (6)	3 (4)	3 (4)	42 (58)	16 (22)	1		Mean: 0.51 SD: 1.69 Range: −3 - 5	Mean: 0.18 SD: 1.49 Range: − 3 - 4
Outings	71	0	2 (4)	13 (29)	6 (13)	19 (42)	5 (11)	2	24	Mean: 1.05 SD: 1.90 Range: −3 – 4	Mean: 0.33 SD: 1.68 Range: − 3 - 3
Visiting	69	1 (2)	1 (2)	4 (8)	8 (17)	31 (65)	3 (6)	1	20	Mean: 0.98 SD: 1.66 Range: −3 – 4	Mean: 0.49 SD: 1.38 Range: − 3 - 3
Home	96	1 (1)	0	1 (1)	7 (7)	68 (71)	19 (20)	0		Mean: 0.18 SD: 1.17 Range: −4 - 4	Mean: 0.11 SD: 1.12 Range: − 4 - 4
Independence	106	0	2 (2)	8 (8)	14 (13)	65 (61)	17 (16)	0		Mean: 0.79 SD: 2.45 Range: −2 – 5	Mean: 0.45 SD: 1.39 Range: −3 - 4
Extra	20	2 (14)	1 (7)	1 (7)	3 (21)	7 (50)	0	1	5	Mean: 0.50 SD: 1.65 Range: −2 - 3	Mean: −0.29 SD: 1.74 Range: − 2 - 2

*SD* = Standard deviation

##### Reliability baseline questions

For the test-retest reliability, 62 participants were approached. In 12 cases, the participant refused the retest, resulting in 50 participants performing a baseline test-retest reliability. In 45 cases the retest was performed by another interviewer and in five cases by the same interviewer.

Of the 21 specified goals from which participants could select, the number of discrepancies between test and retest per participant ranged from zero to a maximum of eleven (52% of the number of goals) with a median of 5 (24%). Of the total of 242 discrepancies, the goal was selected only during the test 87 (36%) times, and in 155 (64%) cases only during the retest.

Item level agreement is included in Additional file 5.

Forty-one retest participants had a follow-up. The PBI_1_ test of the participants who had a baseline retest ranged from − 1.12 to 2.60, with a mean of 0.55 and standard deviation (SD) of 0.83. The PBI_1_ of the retest ranged from − 1.05 to 2.45, with a mean of 0.46 and of SD 0.82. The ICC between PBI_1_ of test and retest was 0.76 (95% CI 0.59;0.86).

The PBI_2_ test of the participants who had a baseline retest ranged from − 1.13 to 2.62, with a mean of 0.56 and SD 0.84. The PBI_2_ of the retest ranged from − 1.00 to 2.45, with a mean of 0.49 and an SD of 0.84. The ICC between PBI_2_ of test and retest was 0.73 (95% CI 0.54;0.85).

##### Reliability follow-up questionnaire

For the follow-up test-retest reliability, 90 participants were approached. In 17 cases the participant refused the retest, six times the participant could not be reached, one participant was sick at the moment of retest and for six it was unknown why the retest was not performed. Finally, 60 participants performed a test-retest of the follow-up questionnaire. Nine participants indicated their situation had changed between test and retest and were removed from analysis, resulting in 51 retests. Median time between test and retest was 7 days. In 36 cases the retest was performed by another interviewer and in 15 cases by the same interviewer.

The agreement on item level is included in Additional file 6.

For the calculation of the PBI, we excluded one case, because only one out of 18 answers of the retest was saved in the computer system. The PBI_1_ test of the participants who had a follow-up retest ranged from − 1.04 to 2.87, with a mean of 0.26 and an SD of 0.70. The PBI_1_ of the retest follow-up ranged from − 1.26 to 2.59, with a mean of 0.27 and an SD of 0.72. The ICC between the PBI_1_ of the test and retest follow-up was 0.86 (95% CI 0.77;0.92).

The PBI_2_ of the participants with a follow-up retest ranged from − 1.00 to 2.91, with a mean of 0.27 and an SD of 0.69. The PBI_2_ of the retest follow-up ranged from − 1.25 to 2.58, with a mean of 0.27 and an SD of 0.71. The ICC between the PBI_2_ of the test and retest follow-up was 0.85 (95% CI 0.76;0.92).

##### Validity importance of goals

All hypotheses, except for hypothesis 11, were confirmed. Table 5 shows the test statistics and complete descriptive information is shown in Additional file 7. The hypothesis for ‘bowel movement’ could not be calculated because of too low cell frequencies.

The 50 cases selected for the open question mentioned 98 goals in total. Of these, 13 goals could not be coded as an item in the P-BAS-P because they did not exist in the P-BAS-P, and were therefore coded as ‘other’. We consequently analysed the agreement between the codes and the answers given in the P-BAS-P of 85 goals and found an agreement of 89%. An overview of the number of items coded and the amount of agreement is given in Table 9.

**Table 9:** Coding of open questions and agreement with P-BAS-P in descending order of frequency

Code	Frequency coded	Agreement n (%)	No agreement n (%)
Curing	18	17 (94)	1 (6)
Other	13	n.a.	n.a.
Alive	8	8 (100)	0
Walking	7	7 (100)	0
Energy	7	7 (100)	0
Hobbies	7	7 (100)	0
Sports	6	5 (83)	1 (17)
Outings	6	4 (67)	2 (33)
Pain	5	3 (60)	2 (40)
Shortness of breath	4	4 (100)	0
Home	4	3 (75)	1 (25)
Independence	3	1 (33)	2 (67)
Knowing what is wrong	3	3 (100)	0
Groceries	2	2 (100)	0
Enjoy	2	2 (100)	0
Better	1	1 (100)	0
Garden	1	1 (100)	0
Visiting	1	1 (100)	0
Driving	0	n.a.	n.a.
Bowel movements	0	n.a.	n.a.
Appetite	0	n.a.	n.a.
Wash and dress	0	n.a.	n.a.
Total	98	76 (89)	9 (11)

The number of confirmed hypotheses regarding importance of goals exceeded the threshold for validity.

##### Validity status baseline, follow-up, and change

As seen in Table 6, all correlations between baseline, follow-up status and the related constructs, are in the direction hypothesised, but from baseline correlations, only four were strong enough and from follow-up six were strong enough to confirm the hypotheses. Of the correlations between change scores, two were strong enough to confirm the hypotheses. For the item ‘sports’, the correlation was in the opposite direction than was hypothesised. As only the minimum of six confirmed hypotheses was reached for the follow-up status, this was the only moment where the status question was considered valid.

##### Responsiveness

Of the 50 cases selected at baseline for comparing open questions, 46 had a follow-up. This resulted in 61 dyads of coded open goals and P-BAS-P items with follow-ups. The correlation between the answers on the open question and the corresponding P-BAS-P score was 0.26 and therefore the hypothesis was rejected.

PBI_1_ ranged from − 1.63 to 2.87, with a mean of 0.31 and an SD of 0.80 and PBI_2_ ranged from − 1.94 to 2.91, with a mean of 0.32 and an SD of 0.81. For the anchor question ‘How much have you benefited from the admission?’ ten (7%) participants did not know what to answer. Of the valid responses, ten (8%) of the participants answered ‘not at all’, five (4%) ‘a little bit’, twenty (15%) ‘somewhat’, 45 (36%) much, and 46 (37%) very much. The Spearman’s correlation coefficient between PBI_1_ and the anchor question was 0.267, between PBI_2_ and the anchor question 0.272.

After coding the explanations participants gave to the anchor question, in ‘based on outcomes’, or ‘based on other grounds’, we found that 101 participants (83%) based their judgements on outcomes, for example ‘I have no longer chest pain’, and 21 (17%) on other grounds, for example ‘top nurses, they were very correct’. Seven participants gave no explanation. In a selection of participants basing their judgement on outcomes, PBI_1_ ranged from − 1.63 to 2.60, with a mean of 0.38 and a standard deviation of (SD) 0.79 and PBI_2_ ranged from − 1.94 to 2.62, with a mean of 0.38 and an SD of 0.80. The correlation between PBI_1_ and anchor question was 0.376 and the correlation between PBI_2_ and anchor question was 0.389.

##### Interpretability

The visual anchor-based minimal important change distribution method was based on the selection of participants basing their judgement on outcomes.

**Fig. 4:**
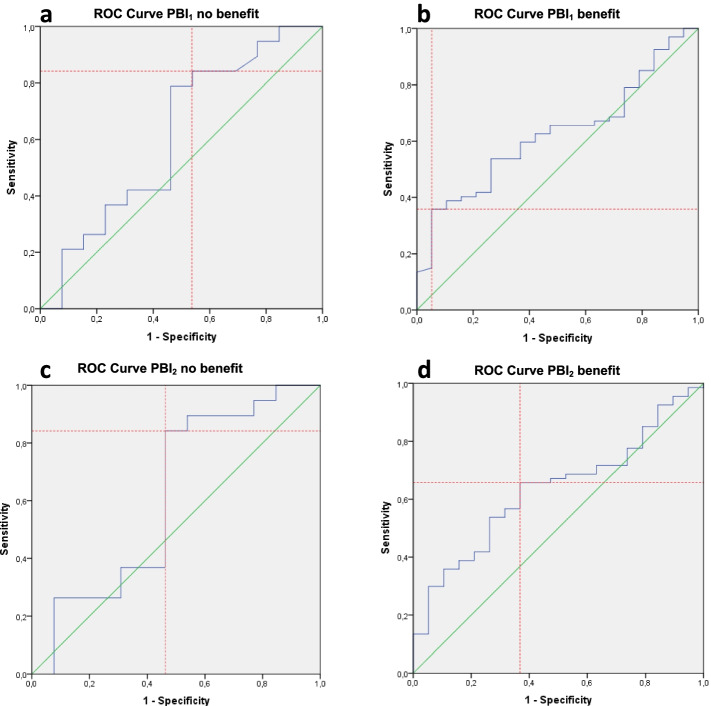
ROC curve with optimal cut-off point. **a** PBI_1_‘no benefit’ (*n* = 32, AUC = 0.61). **b** PBI_1_ ‘benefit’ (*n* = 86, AUC = 0.63). **c** PBI_2_ no benefit’ (*n* = 32, AUC = 0.62). **d** PBI_2_ ‘benefit’ (*n* = 86, AUC = 0.63). ROC = receiver operating characteristic, PBI = Patient Benefit Index, AUC = Area under de curveThe anchor-based MIC distribution is displayed in Fig. 5. As visualised in Fig. 5, the SDC is larger than the MIC, especially for PBI_2._ There is much overlap between the curves, leading to much misclassification

The upper half of Fig. 4 shows ROC curves of PBI_1_ with the ROC curve of ‘no important benefit’ on the left side, with an area under the curve (AUC) of 0.61. The optimal cut-off point for ‘no important benefit’ was set at a sensitivity value of 84% and a specificity of 46%, resulting in an MIC of − 0.3 points on the PBI_1_. The right side shows the ROC curve of ‘important benefit’, with an AUC of 0.63. The optimal cut-off point for ‘important benefit’ was set at a sensitivity value of 36% and a specificity of 95%, resulting in an MIC of 0.9 points on the PBI_1_. This means PBI_1_ values between − 0.3 and 0.9 points are considered as ‘borderline benefit’.

The lower half of Fig. 4 shows the ROC curves of PBI_2_ with the ROC curve of ‘no important benefit’ on the left side, and an AUC of 0.62. The optimal cut-off point for ‘no important benefit’ was set at a sensitivity value of 84% and a specificity of 54%, resulting in an MIC of − 0.3 points on the PBI_2_. The right side shows the ROC curve of ‘important benefit’, with an AUC of 0.63. The optimal cut-off point for ‘important benefit’ was set at a sensitivity value of 66% and a specificity of 66%, resulting in an MIC of 0.2 points on the PBI_2_. This means the PBI_2_ values between − 0.3 and 0.2 are considered as ‘borderline benefit’.

The SEM for PBI_1_ was 0.41, resulting in an SDC of 1.1. The SEM for PBI_2_ was 0.44, resulting in an SDC of 1.2.

## Discussion

In this study, we described the development, feasibility, reliability, validity, responsiveness, and interpretability of the Patient Benefit Assessment scale Picture version (P-BAS-P), a modified version of the P-BAS HOP, which was designed to identify the goals of the individual patient and to measure his relevant outcomes. The results are mixed.

### Feasibility

The baseline pilot and field tests revealed that the P-BAS-P is feasible, but requires good interviewer’s instructions. Some participants needed only a short introduction and little guidance from the interviewer, whereas others needed constant guiding and remembering of the aim. The pictures were considered helpful by the participants. A review recommended that different viewers may interpret pictures differently, so guiding is always needed with these kinds of tools [12].

The fact that many interviews were not conducted on the day of admission was potentially problematic, since the (health) status of participants changed considerably and participants had to recall their situation as it was earlier on the day of admission. For the follow-up, we chose format one, where participants were asked how their status per item was at that moment since the pilot revealed this was easily understood.

When comparing the baseline descriptive statistics of the P-BAS-P with the earlier version of the P-BAS HOP [10], some remarkable differences are seen. With the P-BAS-P more goals are selected at minimum ‘somewhat important’: in the P-BAS HOP, the median number of goals selected by participants was five, with 11 participants not selecting any goal. In the current P-BAS-P, the median number of goals selected was 12 with a minimum of three goals selected per participant. It seems that the threshold to selecting a goal is lowered by differentiating between prevention, preservation, and improvement, and by leaving out the first step in which participants are asked whether they were experiencing difficulty with a subject. Participants might therefore select goals which were not relevant for them, although in the P-BAS HOP participants often stated in the follow-up that a certain goal was not applicable for them, while this was not the case in the current P-BAS-P. Other differences between descriptive statistics of the two versions are that the current P-BAS-P shows more diversity in importance items and an extra goal is mentioned more frequently (28 out of 169 versus 19 out of 451). This could mean that participants are more involved with the P-BAS-P because they have to place the cards on the answer sheets themselves, instead of just answering questions given by an interviewer. This is in line with other research where was found that patients were more likely to read text when pictures were added or patients were more engaged by the inclusion of pictures [12, 26].

### Reliability

The ICC of the PBI is acceptable, with the ICC on follow-up being more satisfactory. This is because when calculating the PBI for test and retest at follow-up, only the status varies; while at baseline, the status, as well as the importance and the choice between prevention/preservation and improvement, vary. Moreover, participants had to remember their health status often from 2 to 4 days prior and are assumed to be in a more unstable situation during baseline than follow-up.

When examining the reliability at the item level (Additional files 3 and 4), a broad variation in kappa values is seen, with the reliability of status being highest, especially at follow-up and of the choice between prevention/preservation and improvement being lowest. This suggests that the question about status is more easily understood, or is least ambiguous to explain by the interviewer. A change in the choice between prevention/preservation and improvement is often caused by a change in status. For example, a participant said during the test that the status of an item was ‘good’ and chose preservation, while stating at retest it was ‘mediocre’, and chose improvement. This makes the test-retest disagreement more logical.

**Fig. 5:**
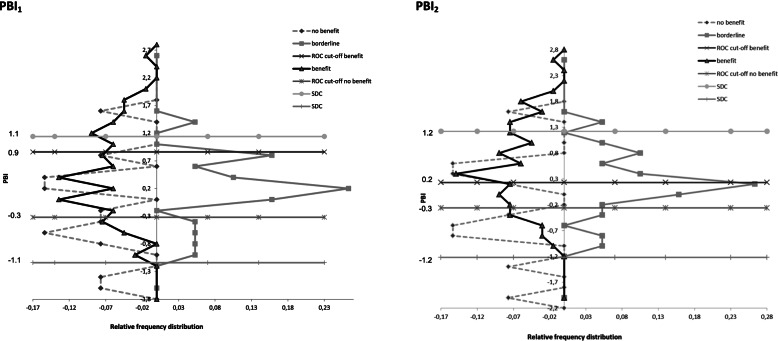
Anchor-based minimal important change (MIC) distributions. Figure left: PBI_1_, figure right: PBI_2_. On left side of figures distribution of participants classified as having relevant benefit benefit (dark line) and no benefit (grey, dashed line) and right side borderline benefit. Cut-off values represent MIC values and SDC. PBI = Patient Benefit Index, SDC = Smallest Detectable Change

The baseline retest ICC of the P-BAS-P is comparable with the former P-BAS HOP (0.76 (PBI_1_)/0.73 (PBI_2_) versus 0.77) [10], but in the former version only the importance of goals varied, while in this version the choice between prevention/preservation/improvement and the status also varied. In contrast, the follow-up retest ICC has improved from 0.62 to 0.86/0.85 between the two versions, suggesting that the adaptations were an improvement for the reliability. Unfortunately, the reliability of the baseline retest had a large impact on the SEM and consequently, the SDC. This is clearly visible in Fig. 5: 82% of the PBI_1_-values and 84% of the PBI_2_-values fall within the measurement error, and the SDC is far beyond the MIC values.

### Validity

Validity data showed that the selection of goals was logically based on the impairments participants had. However, there are two signs that, in rare cases, the selection of goals was not logical. The first is that some participants indicated not having a problem or limitation with a subject, but mentioned as a goal to improve that subject. The second is that, in very rare cases, participants stated never having done an activity, such as sporting, but selected this goal.

The data of choice between prevention/preservation or improvement provide two discussion points. First, although the relationships between status and the choice between prevention/preservation or improvement were very strong (data not shown), every goal has from one to seven cases of a status judged as (very) good, but with the goal to improve that status, this gives little to no room for improvement, and has therefore consequences for the validity of the scores. Second, the goal ‘return back home’ was the only goal where the choice between preservation or improvement was not directly asked to the participant, but depended on the situation from where the participant was admitted and where he/she wanted to return to (see Additional file 1). The fact that all 11 participants where the choice ‘improvement’ was made were living independently, means that the instruction here was not clear to all interviewers.

The validity of the questions regarding ‘status’ is problematic. In the hypothesised strengths of relationships was already accounted for the fact the P-BAS-P status and other constructs at follow-up both refer to the moment of interview, while at baseline the P-BAS-P asks for the status on the day of admission and the others constructs the moment of interview, which is usually a few days later. Almost all follow-up hypotheses were confirmed, but only half of the baseline hypotheses. Possibly the error caused by different time moments at baseline was larger than expected and this could also have influenced the strength of the relationships of change values. In addition, the unstable situation at baseline could have made the construct assessment more difficult than at follow-up. For exercise or sports, correlations at baseline and follow-up were weak and change scores were in the opposite direction than hypothesised. The related construct was physical activity for at least 30 min; this could be any activity, for example going for a walk, but these activities are not always considered as exercise or sports.

### Responsiveness

The correlations between the answers on the open question on follow-up and the corresponding P-BAS-P scores were too weak to support the validity of the scores. When we computed the SDC for only score, so with maintaining the importance weights constant, the SDC was 1.09. Since most P-BAS-P scores were between − 1 and 1, most fell within the measurement error, which makes the comparison biased. Another explanation as to why the comparison is complicated is that the scales are very different. Even when we leave out all the cases with contrary and half congruent answers (Additional file 8), the correlation would be 0.47. This is because a ‘completely’ attained goal can have a score varying from 0 to 3, which gives rise to doubts about whether the score always reflects the amount of perceived benefit. The goal ‘remaining alive’, for example, has a standard score of 0, but as the mean of the scores is 0.31, the consequence is that having set the goal ‘remaining alive’, means for most participants that the total PBI is lowered. Although this goal is very important for many people, it is difficult to express into a score and therefore we recommend to leave this specific goal out of the calculation of the PBI.

We compared all answers on the selected open goals and answers on the P-BAS-P (Additional file 8) and found some contradictory answers. This could be explained by the aforementioned problems with baseline validity due to the baseline interview taking place a few days after admission. For example, participants with the goal ‘curing’ mentioning at baseline that their disease state was ‘(very) good’, which not only gave no or little room for improvement but also questions the validity of the answer ‘(very) good’ at baseline since it is questionable if the cure was even needed. Another explanation could be response shift. An event like a hospitalisation causes a response shift; participants make different cognitive appraisals at different moments, resulting in a recalibration, reprioritisation, and reconceptualisation of goals [27–29]. In this study, we used absolute scores for the P-BAS-P (‘what is the status now’) and relative comparison for answering the goal in the open question, where the participant (implicit) has to compare the situation now with how it was at baseline [1]. These different comparisons require different cognitive processes and therefore cause different forms of response shift. In addition, relative comparisons are more susceptible to recall bias and social desirability [30].

This pattern also applies to the comparison of the PBI with the anchor question. The correlation between the PBI and anchor question was medium and too low to be a valid anchor. Weak correlations between anchor and change scores are common [31]. According to Cohen [22], correlations in behavioural sciences are rarely high. For this reason, Revicki et al. [32] recommend a correlation of at least 0.30 between anchor and outcome, which we reached, but the price is many misclassifications, as seen in Fig. 5.

Other explanations for the moderate correlations are that a single question, which is the characteristic of an anchor question, is less reliable and valid than a multi-item instrument and response shift, especially the phenomenon that the ratings on the anchor correlate more with the follow-up score than with the change score [31, 33, 34]. These findings in the literature are also supported by the explanations to the anchor question given by the participants. When the participant mentioned a concrete attained outcome as an explanation of the amount of benefit, this was usually only one outcome; in almost half of the cases a disease-specific outcome, such as ‘infection cured’, or ‘tumour completely removed’. In most cases, this correlated with the selected goal and score on the corresponding goal. However, participants always selected multiple goals in the P-BAS-P and the scores on the other goals also count in the PBI, while these are not included in the appraisal of the anchor question. The question arises whether the amount of benefit is too complex to capture in only one question or whether the P-BAS-P takes into account too many items that are not relevant for the participant because, in the end, everything turns on whether the disease ameliorated or not and all the other goals that were selected by the participant were selected because the participant was primed by the pictures, but this was not what really mattered. It could also be that the participant gave socially desirable answers, since a hospital is a medical environment, participants judge the benefit of hospitalisation in medical terms because they might think this is expected. Examples of response shift are also seen. For example, one participant stated that he had ‘a little bit of benefit’ from the hospital admission because he declared that his shortness of breath worsened due to the admission, while according to the P-BAS-P the shortness of breath ameliorated from ‘very bad’ to ‘mediocre’. This is an example of the anchor correlating more with the follow-up answer than the change score.

Another discussion point is the anchor question itself and its answer options. We asked for the amount of benefit on a scale from ‘not at all’ to ‘very much’. Most similar studies use an anchor in which participants can distinguish between no change, several grades of improvement, and several grades of deterioration [13, 24, 31, 34]. This means that we had ‘not at all’ as the bottom line, while ignoring the options of deterioration, while the current PBI, in contrast to the former version, does give negative values.

PBI_2_ has a slightly better correlation with the anchor question than PBI_1_, suggesting that the importance of goals counts for the perceived benefit. This is confirmed by the fact that the correlation was lowest when goals were not weighted, with a correlation of 0.259 for the whole sample and 0.362 for the selection of participants basing their judgment on outcomes. However, the consequence is lower reliability since a deviation of importance of goals has more impact on the variation of the PBI_2_.

The PBI of the former P-BAS-HOP, which was identical to follow-up format two of the current version, which we abandoned, had a correlation of 0.51 with the anchor question and therefore was considered valid [10]. This could be due to the fact that the follow-up questions, used to calculate the PBI, and the anchor question were more comparable, using relative comparison and the answer scales were almost identical. Nevertheless, we still think we had good arguments to abandon format two, since the pilot revealed that, in contrast to format one, both the sentences of the follow-up questions were considered too complicated and the timeframes were unclear. We could have tried to specify the timeframe in the questions, but that would have made the questions even more complex. Moreover, other research showed that even when timeframes are specified, these frames were rarely used, on the contrary, participants used for them meaningful timeframes [30].

### Comparison with other tools

Indicating individual priorities is also possible with for example the Goal Attainment Scaling (GAS), but the GAS is more time consuming, varying from 15 to 20 min per participant for experienced assessors [35], to 90 min [36], while the P-BAS-P takes on average 11 min per participant. Moreover, for some older adults it might be difficult to formulate their own goals [37], and the P-BAS-P helps with examples of predefined goals. Other tools are only suitable for specific activities, such as the Canadian Occupational Performance Measure (COPM) [38–40], Self-Identified Goals Assessment (SIGA) [40], Assessment of Motor and Process Skills (AMPS) [38]. In contrast, the P-BAS-P covers many dimensions like disease-related, complaints, daily and social functioning, and could therefore replace a diversity of existing tools.

### Limitations

The main limitation was that the first interview usually took place a few days after admission, sometimes even on the same day as discharge. It is not only unnatural to discuss goals in a late stadium of the admission, it also might have hampered the feasibility, reliability, validity, and responsiveness.

Due to the Corona pandemic, we had to stop the inclusion of new participants. Therefore the sample size is lower than we opted for. Although 136 complete cases are sufficient for the analyses we used, it appeared sometimes to be too few when we analysed on a per-item level. Furthermore, most follow-ups were conducted while we had Corona-measures like staying at home, avoiding visiting other people, and places where many people gather were closed. This made the answering of some questions, especially those about sports, outings, and visiting complicated and probably biased. We placed missing values in cases where participants placed Corona-related remarks, but in cases where participants answered the questions without remarks, the answers were possibly still biased.

Missing values at baseline were caused by the interviewer needing to circle the outcomes on paper while removing the cards, which is sensitive to errors and omissions.

We had a fluctuating team of 13 interviewers, which not only required significant effort to train and familiarise them in the use of the P-BAS-P, but could also lead to undesirable variation between interviewers. The time between discharge and follow-up was 3 months, which might be quite long if patients have to indicate the benefit of hospitalisation.

## Conclusion and recommendations

The P-BAS-P appeared to be a feasible instrument, but there were methodological barriers for the evaluation of the reliability, validity, and responsiveness. Lessons learned from this process are that developing a new tool is a very intensive, time consuming and iterative process, context and timing are very important in testing and evaluating these kind of tools and change scores are difficult to interpret due to measurement error, recall bias, and response shift. We, therefore, recommend further research into the feasibility, reliability, validity, and responsiveness of the P-BAS-P with the first interview preferably before hospitalisation, or, in case of an acute hospital admission, on the first day. Because of the hampered change scores, we recommend showing participants their baseline answers again at follow-up [41]. To evaluate the responsiveness, we recommend a broader anchor question in which participants can distinguish between no change, several grades of improvement, and several grades of deterioration [34].

## Supplementary Information

**Additional file 1.** Description of P-BAS-P.

**Additional file 2.** Three-Step Test-Interview (TSTI).

**Additional file 3.** Questionnaires used to test the construct validity.

**Additional file 4.** Other questionnaires. Baseline, Follow-up and Change scores.

**Additional file 5.** Test-retest baseline item level.

**Additional file 6.** Test-retest Follow-up on item level.

**Additional file 7.** Crosstabulations validity hypotheses baseline importance of goals.

**Additional file 8.** Comparison achievement of goals stated in open question and P-BAS-P scores.

## Data Availability

The datasets used and/or analysed during the current study are available from the corresponding author on reasonable request.

## Abbreviations

ADL: Activities of Daily Living
AUC: Area Under the Curve
ICC: Intraclass Correlation Coefficient
MIC: Minimal Important Change
NRS: Numeric Rating Scale
P-BAS HOP: Patient Benefit Assessment Scale for Hospitalised Older Patients
P-BAS-P: Patient Benefit Assessment Scale Picture Version
PBI: Patient Benefit Index
ROC: Receiver Operating Characteristic
RSCL: Rotterdam Symptom Checklist
SF-36: 36-Item Short Form Survey Instrument
S: Score
SD: Standard Deviation
SDC: Smallest Detectable Change
TSTI: Three-Step Test-Interview
VAS: Visual Analogue Scale
VMS: Safety Management Programme
W: Weight
