# Supplementary material for: Adaptation of the Patient Benefit Assessment Scale for Hospitalised Older Patients: development, reliability and validity of the P-BAS picture version
Source: BMC Geriatr. 2022 Jan 11;22:43. doi: 10.1186/s12877-021-02708-7 (PMC8751090; doi:10.1186/s12877-021-02708-7)
Supplement: Supplementary file 1 — Additional file 1. Description of P-BAS-P. [file 12877_2021_2708_MOESM1_ESM.docx]

**Additional file 1. Description of P-BAS-P**

**Adaptation of the Patient Benefit Assessment Scale for Hospitalised Older Patients: development, reliability and validity of the P-BAS Picture version**

**Authors:**

1. Maria Johanna van der Kluit, MSc RN (Corresponding author)

University of Groningen, University Medical Center Groningen, University Center for Geriatric Medicine, Hanzeplein 1, 9700 RB Groningen, The Netherlands

[m.j.van.der.kluit@umcg.nl](mailto:m.j.van.der.kluit@umcg.nl)

+31503613921

1. Geke J. Dijkstra, PhD

University of Groningen, University Medical Center Groningen, Department of Health Sciences, Applied Health Research, Groningen, The Netherlands

NHL Stenden University of Applied Sciences, Research Group Living, Wellbeing and Care for Older People, Leeuwarden, The Netherlands

[g.j.dijkstra@umcg.nl](mailto:g.j.dijkstra@umcg.nl)

1. Sophia E. de Rooij, MD PhD

University of Groningen, University Medical Center Groningen, University Center for Geriatric Medicine, Groningen, The Netherlands

[sejaderooij@gmail.com](mailto:sejaderooij@gmail.com)

**Additional file 1. Description of P-BAS-P**

**List of goals/ card subjects:**

1. Feeling better/feeling good

2. Energy

3. Pain

4. Bowel movements

5. Shortness of breath

6. Walking

7. Appetite

8. Knowing what is wrong with me

9. Curing/controlling my disease

10. Remaining alive

11. Enjoying life

12. Groceries

13. Washing and dressing

14. Gardening

15. Exercise or Sports

16. Hobbies

17. Driving

18. Going on outings

19. Visiting

20. Return back to my home

21. Independence

22. Extra option

**User Guide**

**Preparations:**

*Place the two different answer sheets in front of the participant on the (bed) table. Make sure that the cards are in the correct order, always two identical cards together, with the exception of ‘remaining alive’ (only one card). Give the cards to the participant (if possible).*

**Instruction text:**

*Don't explain all steps (it is too much information).*

The following questions are about the goals you have with this hospitalisation. A goal is something you want to achieve with this hospitalisation.

These cards show example goals such as alleviating or preventing certain complaints or maintaining or improving your functioning. You can indicate per goal whether this is applicable to you.

If the goal is not applicable to you or you don’t find it important, place the card (2 pieces) on this box: ‘does not apply/not at all important’*(indicate)*. But when a card is a goal for you, I will help you with the next steps.

**Further instructions for the interviewer**

Help with the first 2 goals by default:

- Is ‘feeling better’ a goal for you with this hospitalisation?
- Is maintaining or improving your energy a goal for you with this hospitalisation?

If the participant understands the question ‘is this a goal with this hospitalisation?’, he/she can independently select/place cards, but always help with the follow-up questions. If the participant forgets the question, then keep repeating: Is [subject] a goal for you with this hospitalisation?

If the subject is a goal (order may vary):

- Ask how [subject] was going on the day of admission (very bad, bad, mediocre, satisfactory, good, very good), indicate on the answer sheet
- Ask whether it concerns prevention/preservation or improvement
- Ask how important this goal is (somewhat, quite, very), indicate on the answer sheet
- Place the cards on the correct boxes with the correct side up.

**Regarding ‘prevention/preservation of improvement’:**

- Symbols:
  - =, blue side: prevention (symptoms)/preservation (all other subjects)
  - ↑ , green side**:** improvement
  - Yellow side: prevention/preservation/improvement is not applicable. This concerns ‘knowing what is wrong with me’ and ‘remaining alive’.
- Sometimes the participant indicates himself which one applies (for example*: I want to be able to continue exercising; or pain, that’s important that it decreases*), if this is not clear: ask.
- For symptoms, assume 'improvement', sometimes 'prevention' will be relevant (for example a preventive surgery). Use ‘improving’ or ‘preservation’ for all the other subjects.

Exceptions/special cases:

**Feeling better/ feeling good**

Start with the goal ‘feeling better’. Sometimes a participant will indicate he/she is feeling good, but wanting to keep it that way, then give the option ‘feeling good’.

**Curing/controlling my disease**

Make the participant choose one of two. If ‘curing’ is not possible, controlling disease process is perhaps applicable?

Ask how the disease/condition is going now (very bad, bad, mediocre, satisfactory, good, very good).

It is also possible that the participant considers neither side important.

**Remaining alive**

This goal has only one card.

Do not ask about bad/mediocre/satisfactory/good.

Only ask for importance.

**Return back to my home**

When someone is admitted from a home situation and aims to return to that same home situation, indicate as prevention/preservation.

When someone is admitted from, for example, a (temporary) nursing home or rehabilitation centre and the goal is returning to their own (care) home, indicate improvement.

Ask how it is going with living at home now/ on the day of admission (very bad, bad, mediocre, satisfactory, good, very good).

**?**

Ask for an additional goal. All standard questions apply. Write down the goal in box 22 of the score sheet.

**Regarding ‘How is it going with…’**

When the interview takes place on the day of admission, ask about the current situation. When the participant has been admitted for a few days, it is possible that goals have already ameliorated or been achieved at the moment of interview. In that case, ask how the situation was on the day of admission. The question about prevention/preservation or improvement, starts from the situation on the moment of admission.

If the participant has difficulty indicating how it is going with, for example, the groceries, because he/she is in hospital, ask how it is going ‘in the condition you were on the day of admission’ or ‘in your current condition’.

Examples questions ‘how is it going now with… / how was it on the day of admission with…’:

| Card | Question ‘how does/ did….’ |
| --- | --- |
| 1. Feeling better/ feeling good | How did you feel on the day of admission? |
| 2. Energy | How did your energy go on the day of admission? |
| 3. Pain | How did your pain go on the day of admission? |
| 4. Bowel movements | How did your bowel movements go on the day of admission? |
| 5. Shortness of breath | How did your shortness of breath go on the day of admission? |
| 6. Walking | How did walking go on the day of admission? |
| 7. Appetite | How was your appetite on the day of admission? |
| 8. Knowing what is wrong with me | How was your knowledge about what is wrong with you on the day of admission? |
| 9. Curing/controlling my disease | How was your disease/condition on the day of admission? |
| 10. Remaining alive | n.a. |
| 11. Enjoying life | How did you enjoy life on the day of admission? |
| 12. Groceries | How did the groceries go on the day of admission? |
| 13. Washing and dressing | How did washing and dressing yourself go on the day of admission? |
| 14. Gardening | How did gardening go on the day of admission? |
| 15. Exercise or Sports | How did exercise or sports go on the day of admission? |
| 16. Hobbies | How did the execution of your hobbies go on the day of admission? |
| 17. Driving | How did driving go on the day of admission? |
| 18. Going on outings | How did going on outings go on the day of admission? |
| 19. Visiting | How did visiting go on the day of admission? |
| 20. Return back to my home | How did living at home go on the day of admission? |
| 21. Independence | How did your independence go on the day of admission? |

**Completion**

Score the answers on the score sheet before removing the cards.

Disinfect the cards and answer sheets with OxyWipe tissues, or alcohol 70%. Not with hand alcohol.

**Calculation of the Patient Benefit Index (PBI)**

For the achievement, a Score (S) is calculated based on the difference between status on follow-up and baseline and the choice between prevention/preservation or improvement. The goal is achieved when S = 0. When S > 0, the goal is achieved beyond expectation, when S < 0, the goal is not achieved. In case a goal was prevention or preservation, S = 0 if no change occurred, so follow-up status - baseline status = 0. When the goal was improvement, S = 0 when the status improved, so follow-up status - baseline status = 1. In other words: if the goal was prevention or preservation S = follow-up status – baseline status, if the goal was improvement S = follow-up status – baseline status - 1. The only goal without grades in status is the goal remaining alive. If the participant set that goal, and is alive during follow-up, the participant receives standard Score 0 for that goal.

The PBI is then computed as follows:

$$PBI= \sum_{i=1}^{k} \frac{W_{i}}{\sum_{i=1}^{k} W_{i}} S_{i}$$

with k weight-items ($W_{i}$) and score-items $S_{i}$(range -6 – 5)

For the weight, we tested two weighting schemes to evaluate the role of the importance of the goals in the PBI. Option 1 (PBI_1_): linear: somewhat = 1, quite = 2, very = 3. Option 2 (PBI_2_): quadratic: somewhat = 1, quite = 4, very = 9.

**Score sheet P-BAS-P**

|  | **Goal?** | **Preservation/ Improvement** | | **Score** | | | | | |
| --- | --- | --- | --- | --- | --- | --- | --- | --- | --- |
| 1. **Better/ good** | yes  no | = | ↑ |  |  | somewhat | quite | very |  |
|  |  |  |  | very bad | bad | mediocre | satisfactory | good | very good |
| 1. **Energy** | yes  no | = | ↑ |  |  | somewhat | quite | very |  |
|  |  |  |  | very bad | bad | mediocre | satisfactory | good | very good |
| 1. **Pain** | yes  no | = | ↑ |  |  | somewhat | quite | very |  |
|  |  |  |  | very bad | bad | mediocre | satisfactory | good | very good |
| 1. **Bowel movements** | yes  no | = | ↑ |  |  | somewhat | quite | very |  |
|  |  |  |  | very bad | bad | mediocre | satisfactory | good | very good |
| 1. **Shortness**   **of breath** | yes  no | = | ↑ |  |  | somewhat | quite | very |  |
|  |  |  |  | very bad | bad | mediocre | satisfactory | good | very good |
| 1. **Walking** | yes  no | = | ↑ |  |  | somewhat | quite | very |  |
|  |  |  |  | very bad | bad | mediocre | satisfactory | good | very good |
| 1. **Appetite** | yes  no | = | ↑ |  |  | somewhat | quite | very |  |
|  |  |  |  | very bad | bad | mediocre | satisfactory | good | very good |
| 1. **Knowing what is wrong** | yes  no |  |  |  |  | somewhat | quite | very |  |
|  |  |  |  | very bad | bad | mediocre | satisfactory | good | very good |
| 1. **Curing/ controlling disease** | yes  no | = | ↑ |  |  | somewhat | quite | very |  |
|  |  |  |  | very bad | bad | mediocre | satisfactory | good | very good |
| 1. **Alive** | yes  no |  |  |  |  | somewhat | quite | very |  |
|  |  |  |  |  |  |  |  |  |  |
| 1. **Enjoying** | yes  no | = | ↑ |  |  | somewhat | quite | very |  |
|  |  |  |  | very bad | bad | mediocre | satisfactory | good | very good |

|  | **Goal?** | **Preservation/ Improvement** | | **Score** | | | | | |
| --- | --- | --- | --- | --- | --- | --- | --- | --- | --- |
| 1. **Groceries** | yes  no | = | ↑ |  |  | somewhat | quite | very |  |
|  |  |  |  | very bad | bad | mediocre | satisfactory | good | very good |
| 1. **Washing** | yes  no | = | ↑ |  |  | somewhat | quite | very |  |
|  |  |  |  | very bad | bad | mediocre | satisfactory | good | very good |
| 1. **Gardening** | yes  no | = | ↑ |  |  | somewhat | quite | very |  |
|  |  |  |  | very bad | bad | mediocre | satisfactory | good | very good |
| 1. **Sports** | yes  no | = | ↑ |  |  | somewhat | quite | very |  |
|  |  |  |  | very bad | bad | mediocre | satisfactory | good | very good |
| 1. **Hobbies** | yes  no | = | ↑ |  |  | somewhat | quite | very |  |
|  |  |  |  | very bad | bad | mediocre | satisfactory | good | very good |
| 1. **Driving** | yes  no | = | ↑ |  |  | somewhat | quite | very |  |
|  |  |  |  | very bad | bad | mediocre | satisfactory | good | very good |
| 1. **Outings** | yes  no | = | ↑ |  |  | somewhat | quite | very |  |
|  |  |  |  | very bad | bad | mediocre | satisfactory | good | very good |
| 1. **Visiting** | yes  no | = | ↑ |  |  | somewhat | quite | very |  |
|  |  |  |  | very bad | bad | mediocre | satisfactory | good | very good |
| 1. **Home** | yes  no | = | ↑ |  |  | somewhat | quite | very |  |
|  |  |  |  | very bad | bad | mediocre | satisfactory | good | very good |
| 1. **Independence** | yes  no | = | ↑ |  |  | somewhat | quite | very |  |
|  |  |  |  | very bad | bad | mediocre | satisfactory | good | very good |
|  | yes  no | = | ↑ |  |  | somewhat | quite | very |  |
|  |  |  |  | very bad | bad | mediocre | satisfactory | good | very good |
