# Supplementary material for: Adaptation of the Patient Benefit Assessment Scale for Hospitalised Older Patients: development, reliability and validity of the P-BAS picture version
Source: BMC Geriatr. 2022 Jan 11;22:43. doi: 10.1186/s12877-021-02708-7 (PMC8751090; doi:10.1186/s12877-021-02708-7)
Supplement: Supplementary file 2 — Additional file 2. Three-Step Test-Interview (TSTI). [file 12877_2021_2708_MOESM2_ESM.docx]

**Additional file 2. Three-Step Test-Interview (TSTI)**

**Adaptation of the Patient Benefit Assessment Scale for Hospitalised Older Patients: development, reliability and validity of the P-BAS Picture version**

**Authors:**

1. Maria Johanna van der Kluit, MSc RN (Corresponding author)

University of Groningen, University Medical Center Groningen, University Center for Geriatric Medicine, Hanzeplein 1, 9700 RB Groningen, The Netherlands

[m.j.van.der.kluit@umcg.nl](mailto:m.j.van.der.kluit@umcg.nl)

+31503613921

1. Geke J. Dijkstra, PhD

University of Groningen, University Medical Center Groningen, Department of Health Sciences, Applied Health Research, Groningen, The Netherlands

NHL Stenden University of Applied Sciences, Research Group Living, Wellbeing and Care for Older People, Leeuwarden, The Netherlands

[g.j.dijkstra@umcg.nl](mailto:g.j.dijkstra@umcg.nl)

1. Sophia E. de Rooij, MD PhD

University of Groningen, University Medical Center Groningen, University Center for Geriatric Medicine, Groningen, The Netherlands

[sejaderooij@gmail.com](mailto:sejaderooij@gmail.com)

**Additional file 2. Three-Step Test-Interview (TSTI)**

The TSTI consists of the following steps (1,2):

Step 1: Concurrent thinking aloud. The participant performed version 1 of the P-BAS-P with help of an interviewer while thinking aloud. The observer observed, made notes of the participant’s behaviour (hesitations, corrections) and verbalised thoughts. However, the observer neither spoke nor intervened.

Step 2: Retrospective interview. The observer took over the interviewer role and aimed to fill in any gaps from the first step. Every behaviour and thought from the observation which the interviewer wanted further information about, was clarified.

Step 3: Semi-structured interview. An in-depth interview was conducted, aimed at eliciting participant’s considerations and opinions. The participant was given an opportunity to explain behaviour, actions, or thoughts that he had in previous steps. The participant was asked his opinion about the tool and to explain his goals in his own words in order to perform a first content validation of the P-BAS-P.

**References**

(1) Pool JJM, Hiralal S, Ostelo, Raymond W J G, van der Veer K, de Vet HCW. Added value of qualitative studies in the development of health related patient reported outcomes such as the Pain Coping and Cognition List in patients with sub-acute neck pain. Man Ther 2010;15(1):43-47.

(2) Van Der Veer K, Ommundsen R, Hak T, Larsen KS. Meaning Shift of Items in Different Language Versions. A Cross-National Validation Study of the Illegal Aliens Scale. Quality and Quantity 2003;37(2):193-206.
