# Supplementary material for: Adaptation of the Patient Benefit Assessment Scale for Hospitalised Older Patients: development, reliability and validity of the P-BAS picture version
Source: BMC Geriatr. 2022 Jan 11;22:43. doi: 10.1186/s12877-021-02708-7 (PMC8751090; doi:10.1186/s12877-021-02708-7)
Supplement: Supplementary file 3 — Additional file 3. Questionnaires used to test the construct validity. [file 12877_2021_2708_MOESM3_ESM.docx]

**Additional file 3. Questionnaires used to test the construct validity**

**Adaptation of the Patient Benefit Assessment Scale for Hospitalised Older Patients: development, reliability and validity of the P-BAS Picture version**

**Authors:**

1. Maria Johanna van der Kluit, MSc RN (Corresponding author)

University of Groningen, University Medical Center Groningen, University Center for Geriatric Medicine, Hanzeplein 1, 9700 RB Groningen, The Netherlands

[m.j.van.der.kluit@umcg.nl](mailto:m.j.van.der.kluit@umcg.nl)

+31503613921

1. Geke J. Dijkstra, PhD

University of Groningen, University Medical Center Groningen, Department of Health Sciences, Applied Health Research, Groningen, The Netherlands

NHL Stenden University of Applied Sciences, Research Group Living, Wellbeing and Care for Older People, Leeuwarden, The Netherlands

[g.j.dijkstra@umcg.nl](mailto:g.j.dijkstra@umcg.nl)

1. Sophia E. de Rooij, MD PhD

University of Groningen, University Medical Center Groningen, University Center for Geriatric Medicine, Groningen, The Netherlands

[sejaderooij@gmail.com](mailto:sejaderooij@gmail.com)

**Additional file 3. Questionnaires used to test the construct validity**

Details of the constructs and questionnaires as summarised in Table 2.

**Dutch VMS screening program (VMS)**

The VMS questionnaire, which is developed as part of the Dutch Safety Management Programme consists of four instruments: Activities of daily living (ADL), falls, undernutrition, and delirium. For the hypotheses to test the validity only the question about appetite is analysed: The patient is asked whether he experienced a decrease of appetite during the last month (yes/no) (1). The questions were asked at baseline and follow-up.

**Rotterdam Symptom Checklist (RSCL)**

The RSCL was developed to measure symptoms reported by cancer patients participating in clinical research. It consists of a broad list of symptoms concerning psychological and physical distress (2). Originally, the symptoms are on a four point Likert scale, but we dichotomised the symptoms in present or absent on admission day.

**Pain and Fatigue Numeric Rating Scale (NRS)**

Participants were asked to rate their pain and fatigue as experienced at the moment of the interview. The scale runs from 0: no pain/fatigue at all to 10: the worst imaginable pain/fatigue.

**EQ-5D**

The EQ-5D is a standardised non-disease-specific instrument for describing and valuing health-related quality of life. It consists of five dimensions and a Visual Analogue Scale (VAS). The dimensions are mobility, self-care, usual activities, pain/discomfort, and anxiety/depression, with three answer options each: no problems, some problems and extreme problems. The VAS, often referred to as the EuroQol ‘thermometer’ has an endpoint of 100 for the best imaginable health state and 0 for the worst imaginable health state (3). Participants were asked to indicate their health state on the day of the interview.

**Admission reason**

Admission reason was obtained from the medical record, recorded by the attending physician and retrieved by a medical student. Options were acute/elective; diagnostic/curative/palliative. When a participant gave no informed consent for record insight, this was labelled as ‘unknown’.

**Physical activity**

How often are you physically active for at least 30 minutes? For example having a walk, cycling or swimming. Options were, (hardly) never, monthly, weekly, three times a week, daily. Participants were asked, during the baseline interview, to indicate their frequency two weeks prior to hospital admission and, during the follow-up interview, to indicate their current frequency.

**36-Item Short Form Survey Instrument (SF-36) – Social functioning**

The question ‘During the past 4 weeks, how much of the time has your physical health or emotional problems interfered with your social activities (like visiting with friends, relatives, etc.)?’, is part of the SF-36 Health Survey, but is used as a single item in our survey. The answer options were: none of the time, a little of the time, some of the time, most of the time, all of the time (4).

**Goals on hospital admission**

Open question to the participant at baseline: ‘What do you hope to accomplish with this hospitalisation?’ The goal stated by the participant was repeated at follow-up and asked to what extent he had accomplished the goal with the answer options: ‘not at all’, ‘somewhat’, ‘moderately’ ‘quite’, or ‘completely’.

(1) Heim N, van Fenema EM, Weverling-Rijnsburger AW, Tuijl JP, Jue P, Oleksik AM, et al. Optimal screening for increased risk for adverse outcomes in hospitalised older adults. Age Ageing 2015 Mar;44(2):239-244.

(2) de Haes JC, van Knippenberg FC, Neijt JP. Measuring psychological and physical distress in cancer patients: structure and application of the Rotterdam Symptom Checklist. Br J Cancer 1990 Dec;62(6):1034-1038.

(3) Lamers LM, McDonnell J, Stalmeier PF, Krabbe PF, Busschbach JJ. The Dutch tariff: results and arguments for an effective design for national EQ-5D valuation studies. Health Econ 2006 Oct;15(10):1121-1132.

(4) Aaronson NK, Muller M, Cohen PD, Essink-Bot ML, Fekkes M, Sanderman R, et al. Translation, validation, and norming of the Dutch language version of the SF-36 Health Survey in community and chronic disease populations. J Clin Epidemiol 1998 Nov;51(11):1055-1068.
