# Supplementary material for: Adaptation of the Patient Benefit Assessment Scale for Hospitalised Older Patients: development, reliability and validity of the P-BAS picture version
Source: BMC Geriatr. 2022 Jan 11;22:43. doi: 10.1186/s12877-021-02708-7 (PMC8751090; doi:10.1186/s12877-021-02708-7)
Supplement: Supplementary file 4 — Additional file 4. Other questionnaires. Baseline, Follow-up and Change scores. [file 12877_2021_2708_MOESM4_ESM.docx]

**Additional file 4. Other questionnaires. Baseline, Follow-up and Change scores**

**Adaptation of the Patient Benefit Assessment Scale for Hospitalised Older Patients: development, reliability and validity of the P-BAS Picture version**

**Authors:**

1. Maria Johanna van der Kluit, MSc RN (Corresponding author)

University of Groningen, University Medical Center Groningen, University Center for Geriatric Medicine, Hanzeplein 1, 9700 RB Groningen, The Netherlands

[m.j.van.der.kluit@umcg.nl](mailto:m.j.van.der.kluit@umcg.nl)

+31503613921

1. Geke J. Dijkstra, PhD

University of Groningen, University Medical Center Groningen, Department of Health Sciences, Applied Health Research, Groningen, The Netherlands

NHL Stenden University of Applied Sciences, Research Group Living, Wellbeing and Care for Older People, Leeuwarden, The Netherlands

[g.j.dijkstra@umcg.nl](mailto:g.j.dijkstra@umcg.nl)

1. Sophia E. de Rooij, MD PhD

University of Groningen, University Medical Center Groningen, University Center for Geriatric Medicine, Groningen, The Netherlands

[sejaderooij@gmail.com](mailto:sejaderooij@gmail.com)

**Additional file 4. Other questionnaires. Baseline, Follow-up and Change scores**

|  | Baseline  n= 169 | Follow-up  n= 136 | Change (FU –B) |
| --- | --- | --- | --- |
| Item | n (%) | n (%) | Deterioration (d) : n (%)  No change (n): n (%)  Improvement (i): n (%) |
| VMS - Lack of appetite  missing | 43 (25)  0 | 24 (18) | n.a. |
| RSCL - Lack of appetite  missing | 40 (24)  0 | n.a. | n.a. |
| RSCL - Tiredness  missing | 107 (63)  0 | n.a. | n.a. |
| RSCL - Lack of energy  missing | 62 (37)  0 | n.a. | n.a. |
| RSCL – Constipation  missing | 19 (11)  0 | n.a. | n.a. |
| RSCL – Diarrhoea  missing | 13 (8)  0 | n.a. | n.a. |
| RSCL – Shortness of breath  missing | 87 (52)  0 | n.a. | n.a. |
| EQ-5D – Mobility  No problems  Some problems  Confined to bed  missing | 59 (35)  91 (54)  19 (11)  0 | 71 (52)  63 (46)  2 (2)  0 | d -1: 13 (10)  n:80 (59)  i +1: 37 (27)  i +2: 6 (4) |
| EQ-5D – Self-care  No problems  Some problems  Unable  missing | 106 (63)  47 (28)  16 (10)  0 | 108 (79)  25 (18)  3 (2)  0 | d -1: 10 (7)  n: 91 (67)  i +1: 30 (22)  i +2: 5 (4) |
| EQ-5D – Usual activities  No problems  Some problems  Unable  missing | 54 (33)  65 (39)  47 (28)  3 | 86 (63)  42 (31)  8 (6)  0 | d -2: 2 (2)  d -1: 11 (8)  n: 57(43)  i +1: 38 (29)  i +2: 25 (19) |
| EQ-5D – Pain/discomfort  No  Moderate  Extreme  missing | 58 (34)  93 (55)  18 (11)  0 | 57 (42)  70 (52)  9 (7)  0 | d -2: 1 (1)  d -1: 25 (18)  n: 75 (55)  i +1: 29 (21)  i +2: 6 (4) |
| EQ-5D – VAS  missing | Mean: 61.11  SD: 17.79  Range: 0-100  1 | Mean: 66.11  SD: 20.19  Range: 1-100  0 | Mean: 4.67  SD: 23.93  Range: -72 - 80 |
| Fatigue – NRS  missing | Mean: 4.62  SD: 2.52  Range: 0-10  4 | Mean: 3.85  SD: 2.63  Range: 0-9  0 | Mean: -0.83  SD: 3.60  Range: -10 - 8 |
| Pain – NRS  missing | Median: 1  IQR: 0-4  Range: 0-8  5 | n.a. | n.a. |
| SF-36 - Social activities  missing | Median: 1  IQR: 0-2  Range: 0-4  5 | Median: 1  IQR: 0-2  Range: 0-4  20* | Mean: -0.12  SD: 1.54  Range: -4 - 4 |
| Physical activity  missing | Median: 3  IQR: 0-4  Range: 0-4  4 | Median: 4  IQR: 3-4  Range: 0-4  1 | Mean: 0.56  SD: 1.55  Range: -4 - 4 |
| Living situation  Independent  Sheltered accommodation  Senior home  Nursing home  missing | 162 (96)  4 (2)  2 (1)  1 (1)  0 | 133 (98)  0  2 (2)  1 (1)  0 | No change: 133 (98)  To more dependent: 1 (1)  To more independent: 2 (2) |

EQ-5D – VAS: Higher number is better perceived health. Change: positive is increase in perceived health

Fatigue –NRS: numeric rating scale. Higher number is more fatigue. Change: positive is increase in experienced fatigue

Pain–NRS: numeric rating scale. Higher number is more experienced pain.

SF-36: higher number is more experienced limitation. Change: positive is increase in interference with social activities.

SD = Standard deviation, IQR = Interquartile range

* 19 missing values due to Corona
