# Supplementary material for: Adaptation of the Patient Benefit Assessment Scale for Hospitalised Older Patients: development, reliability and validity of the P-BAS picture version
Source: BMC Geriatr. 2022 Jan 11;22:43. doi: 10.1186/s12877-021-02708-7 (PMC8751090; doi:10.1186/s12877-021-02708-7)
Supplement: Supplementary file 5 — Additional file 5. Test-retest baseline item level. [file 12877_2021_2708_MOESM5_ESM.docx]

**Additional file 5. Test-retest baseline item level**

**Adaptation of the Patient Benefit Assessment Scale for Hospitalised Older Patients: development, reliability and validity of the P-BAS Picture version**

**Authors:**

1. Maria Johanna van der Kluit, MSc RN (Corresponding author)

University of Groningen, University Medical Center Groningen, University Center for Geriatric Medicine, Hanzeplein 1, 9700 RB Groningen, The Netherlands

[m.j.van.der.kluit@umcg.nl](mailto:m.j.van.der.kluit@umcg.nl)

+31503613921

1. Geke J. Dijkstra, PhD

University of Groningen, University Medical Center Groningen, Department of Health Sciences, Applied Health Research, Groningen, The Netherlands

NHL Stenden University of Applied Sciences, Research Group Living, Wellbeing and Care for Older People, Leeuwarden, The Netherlands

[g.j.dijkstra@umcg.nl](mailto:g.j.dijkstra@umcg.nl)

1. Sophia E. de Rooij, MD PhD

University of Groningen, University Medical Center Groningen, University Center for Geriatric Medicine, Groningen, The Netherlands

[sejaderooij@gmail.com](mailto:sejaderooij@gmail.com)

**Additional file 5. Test-retest baseline item level**

Percentage of agreement, Cohen’s kappa with quadratic weighting and maximum attainable kappa (1,2) were calculated per item for agreement on importance and status of the goals. For the choice between prevention/preservation or improvement, unweighted kappa values were calculated. Kappa values and confidence intervals were calculated using IBM SPSS 27. Maximal attainable kappa values were calculated using online calculator (3). Kappa values where only calculated when the sample size was >10 participants. For the interpretation of the kappa values, the classification of Landis and Koch (4) was used.

Table 1 shows the weighted kappa values for baseline importance items in descending order. Seven items had substantial agreement, six moderate agreement, five fair agreement and three slight agreement. When the weighted kappa was calculated as a proportion of the maximum attainable kappa, nine items had substantial agreement, eight items moderate agreement, one fair agreement and three slight agreement. The mean of all the weighted kappa values showed moderate agreement.

Table 2 shows the weighted kappa values for baseline status items in descending order. Seven items had substantial agreement, four moderate agreement, and six fair agreement. When the weighted kappa was calculated as a proportion of the maximum attainable kappa, three items had almost perfect agreement, five items had substantial agreement, five items moderate agreement, and three fair agreement. The mean of all the weighted kappa values showed moderate agreement, when calculated as a proportion of the maximum attainable kappa, substantial agreement.

Table 3 shows the kappa values for baseline items prevention/preservation or improvement in descending order. Three items had substantial agreement, three moderate agreement, eight fair agreement and two slight agreement. When the weighted kappa was calculated as a proportion of the maximum attainable kappa, two items had perfect agreement, two items had substantial agreement, seven items moderate agreement, two fair agreement, and two slight agreement. The mean of all the weighted kappa values showed fair agreement when calculated as a proportion of the maximum attainable kappa, moderate agreement.

From the participants that mentioned an extra goal, only two participants named the same goal in the test and retest, with high agreement on all questions. Full crosstabulations of all items are shown in tables 4 – 6.

Table 1. Cohen’s weighted kappa with quadratic weighting for baseline importance items in descending order (n=49-50)

| Item | % agreement | Weighted Kappa (95% CI) | K_max_ | Weighted K/ K_max_ |
| --- | --- | --- | --- | --- |
| Sports | 72.00 | 0.71 (0.51;0.92) | 0.90 | 0.79 |
| Better | 74.00 | 0.65 (0.35;0.94) | 0.85 | 0.76 |
| Shortness of breath | 62.00 | 0.64 (0.44;0.84) | 0.91 | 0.70 |
| Hobbies | 66.00 | 0.63 (0.43;0.83) | 0.86 | 0.73 |
| Driving | 66.00 | 0.63 (0.42;0.83) | 0.97 | 0.64 |
| Garden | 76.00 | 0.61 (0.37;0.85) | 0.88 | 0.69 |
| Outings | 60.00 | 0.61 (0.39;0.82) | 0.99 | 0.61 |
| Pain | 66.00 | 0.55 (0.32;0.78) | 0.75 | 0.74 |
| Walking | 60.00 | 0.50 (0.27;0.74) | 0.98 | 0.51 |
| Bowel movements | 82.00 | 0.48 (0.13;0.82) | 0.70 | 0.69 |
| Wash and dress | 64.00 | 0.44 (0.19;0.68) | 0.77 | 0.57 |
| Energy | 56.00 | 0.44 (0.18;0.71) | 0.81 | 0.55 |
| Appetite | 64.00 | 0.43 (0.16;0.70) | 0.96 | 0.45 |
| Visiting | 58.00 | 0.38 (0.11;0.65) | 0.88 | 0.44 |
| Alive | 66.00 | 0.36 (0.03;0.70) | 0.93 | 0.39 |
| Groceries | 52.00 | 0.36 (0.10;0.62) | 0.82 | 0.43 |
| Enjoy | 55.00 | 0.33 (0.08;0.59) | 0.70 | 0.47 |
| Knowing what is wrong | 61.22 | 0.25 (0.01;0.49) | 0.46 | 0.55 |
| Curing | 66.00 | 0.09 (-0.15;0.32) | 0.78 | 0.11 |
| Home | 53.06 | 0.03 (-0.21;0.28) | 0.48 | 0.07 |
| Independence | 44.00 | -0.01 (-0.24;0.22) | 0.48 | -0.02 |
| Extra (n=2) | 100 | nc | nc | nc |
| Mean | 64.69 | 0.43 | 0.80 | 0.52 |

K= kappa K_max_=maximum attainable kappa CI= Confidence interval nc= not calculated

Table 2. Cohen’s weighted kappa with quadratic weighting for baseline status items in descending order (n=2-47)

| Item | n | % agreement | Weighted Kappa (95% CI) | K_max_ | Weighted K/ K_max_ |
| --- | --- | --- | --- | --- | --- |
| Appetite | 10 | 40.00 | 0.80 (0.59;1.02) | 0.80 | 1 |
| Driving | 17 | 58.82 | 0.75 (0.44;1.06) | 0.93 | 0.80 |
| Walking | 32 | 53.00 | 0.72 (0.53;0.92) | nc | nc |
| Pain | 15 | 60.00 | 0.71 (0.43;0.99) | 0.75 | 0.95 |
| Better | 46 | 56.52 | 0.70 (0.53;87) | 0.90 | 0.78 |
| Wash and dress | 14 | 35.71 | 0.70 (0.52;0.88) | 0.93 | 0.75 |
| Sports | 19 | 57.89 | 0.64 (0.26;1.01) | 0.68 | 0.93 |
| Independence | 26 | 38.46 | 0.59 (0.34;0.84) | 0.80 | 0.56 |
| Groceries | 20 | 40.00 | 0.57 (0.25;0.89) | 0.94 | 0.61 |
| Outings | 22 | 40.91 | 0.54 (0.19;0.89) | 0.99 | 0.55 |
| Shortness of breath | 27 | 55.56 | 0.44 (0.17;0.71) | 0.92 | 0.41 |
| Enjoy | 29 | 31.03 | 0.40 (0.14;0.67) | 0.67 | 0.61 |
| Energy | 38 | 34.21 | 0.38 (0.09;0.67) | 0.64 | 0.59 |
| Home | 28 | 53.57 | 0.37 (-0.2;0.75) | 0.86 | 0.43 |
| Hobbies | 21 | 33.33 | 0.36 (-0.2;0.74) | 0.89 | 0.40 |
| Curing | 47 | 46.81 | 0.29 (0;0.59) | 0.93 | 0.31 |
| Visiting | 18 | 61.11 | 0.25 (-0.25;0.76) | 0.81 | 0.31 |
| Knowing what is wrong | 7 | 28.57 | nc | nc | nc |
| Bowel movements | 4 | 25.00 | nc | nc | nc |
| Garden | 9 | 22.22 | nc | nc | nc |
| Extra | 2 | 50.00 | nc | nc | nc |
| Mean | 21.48 | 43.94 | 0.54 | 0.84 | 0.62 |

K= kappa K_max_=maximum attainable kappa CI= Confidence interval nc= not calculated

Table 3. Cohen’s kappa baseline items prevention/preservation or improvement in descending order (n=2-46)

| Item | n | % agreement | Kappa  (95% CI) | K_max_ | K/ K_max_ |
| --- | --- | --- | --- | --- | --- |
| Sports | 18 | 88.89 | 0.68 (0.29;1.08) | 0.68 | 1 |
| Enjoy | 29 | 82.76 | 0.65 (0.37;0.93) | 0.93 | 0.70 |
| Pain | 15 | 93.33 | 0.63 (-0,1;1.23) | 0.63 | 1 |
| Energy | 38 | 86.84 | 0.54 (0.18;0.89) | 0.91 | 0.59 |
| Walking | 32 | 90.63 | 0.52 (0.05;0.99) | 0.84 | 0.62 |
| Curing | 45 | 84.44 | 0.49 (0.17;0.82) | 0.93 | 0.53 |
| Groceries | 20 | 70.00 | 0.40 (0.01;0.79) | 0.80 | 0.50 |
| Appetite | 10 | 70.00 | 0.35 (-0.24;0.94) | 0.78 | 0.44 |
| Independence | 26 | 65.38 | 0.31 (-0.05;0.66) | 0.77 | 0.40 |
| Better | 46 | 84.78 | 0.28 (-0.10;0.67) | 0.69 | 0.41 |
| Hobbies | 20 | 65.00 | 0.27 (-0.14;0.68) | 0.69 | 0.39 |
| Driving | 17 | 70.59 | 0.27 (-0.18;0.73) | 0.56 | 0.48 |
| Home | 28 | 85.71 | 0.26 (-0.24;0.77) | 0.63 | 0.42 |
| Visiting | 18 | 61.11 | 0.22 (-0.23;0.67) | 0.89 | 0.25 |
| Outings | 22 | 63.64 | 0.16 (-0.27;0.59) | 1 | 0.16 |
| Wash and dress | 14 | 57.14 | 0.14 (-0.35;0.64) | 0.71 | 0.20 |
| Bowel movements | 4 | 75.00 | nc | nc | nc |
| Shortness of breath | 27 | 96.30 | nc | nc | nc |
| Garden | 9 | 88.89 | nc | nc | nc |
| Extra | 2 | 100 | nc | nc | nc |
| Mean | 22 | 79.02 | 0.39 | 0.78 | 0.51 |

K= kappa K_max_=maximum attainable kappa CI= Confidence interval nc= not calculated

Table 4. Crosstabulations of test-retest Baseline Importance

| Item | Test  Retest | Doesn’t apply/ not at all important | Somewhat important | Quite important | Very important | Total | Weighted Kappa (95% CI) |
| --- | --- | --- | --- | --- | --- | --- | --- |
| Better | Doesn’t apply/ not at all important | **1** | 2 | 0 | 0 | 3 | 0.65 (0.35;0.94) |
|  | Somewhat important | 0 | **0** | 1 | 0 | 1 |  |
|  | Quite important | 0 | 0 | **3** | 1 | 4 |  |
|  | Very important | 1 | 0 | 8 | **33** | 42 |  |
|  | Total | 2 | 2 | 12 | 34 | **50** |  |
| Item | Test  Retest | Doesn’t apply/ not at all important | Somewhat important | Quite important | Very important | Total | Weighted Kappa (95% CI) |
| Energy | Doesn’t apply/ not at all important | **3** | 0 | 4 | 1 | 8 | 0.44 (0.18;0.71) |
|  | Somewhat important | 1 | **0** | 2 | 0 | 3 |  |
|  | Quite important | 1 | 0 | **8** | 8 | 17 |  |
|  | Very important | 2 | 0 | 3 | **17** | 22 |  |
|  | Total | 7 | 0 | 17 | 26 | **50** |  |
|  | | | | | | | |
| Pain | Doesn’t apply/ not at all important | **25** | 0 | 2 | 1 | 28 | 0.55 (0.32;0.78) |
|  | Somewhat important | 0 | **0** | 0 | 0 | 0 |  |
|  | Quite important | 2 | 3 | **1** | 1 | 7 |  |
|  | Very important | 5 | 0 | 3 | **7** | 15 |  |
|  | Total | 32 | 3 | 6 | 9 | **50** |  |
|  | | | | | | | |
| Bowel movements | Doesn’t apply/ not at all important | **38** | 0 | 5 | 2 | 45 | 0.48 (0.13;0.82) |
|  | Somewhat important | 0 | **0** | 1 | 0 | 1 |  |
|  | Quite important | 0 | 0 | **0** | 0 | 0 |  |
|  | Very important | 1 | 0 | 0 | **3** | 4 |  |
|  | Total | 39 | 0 | 6 | 5 | **50** |  |
|  | | | | | | | |
| Shortness of breath | Doesn’t apply/ not at all important | **14** | 0 | 1 | 2 | 17 | 0.64 (0.44;0.84) |
|  | Somewhat important | 0 | **0** | 0 | 0 | 0 |  |
|  | Quite important | 4 | 0 | **2** | 7 | 13 |  |
|  | Very important | 2 | 0 | 3 | **15** | 20 |  |
|  | Total | 20 | 0 | 6 | 24 | **50** |  |
|  | | | | | | | |
| Walking | Doesn’t apply/ not at all important | **6** | 1 | 3 | 2 | 12 | 0.50 (0.27;0.74) |
|  | Somewhat important | 0 | **0** | 0 | 0 | 0 |  |
|  | Quite important | 4 | 0 | **5** | 5 | 14 |  |
|  | Very important | 2 | 0 | 3 | **19** | 24 |  |
|  | Total | 12 | 1 | 11 | 26 | **50** |  |

| Item | Test  Retest | Doesn’t apply/ not at all important | Somewhat important | Quite important | Very important | Total | Weighted Kappa (95% CI) |
| --- | --- | --- | --- | --- | --- | --- | --- |
| Appetite | Doesn’t apply/ not at all important | **28** | 0 | 2 | 4 | 34 | 0.43 (0.16;0.70) |
|  | Somewhat important | 2 | **0** | 0 | 0 | 2 |  |
|  | Quite important | 3 | 2 | **1** | 3 | 9 |  |
|  | Very important | 1 | 1 | 0 | **3** | 5 |  |
|  | Total | 34 | 3 | 3 | 10 | **50** |  |
|  | | | | | | | |
| Knowing what is wrong | Doesn’t apply/ not at all important | **24** | 0 | 3 | 12 | 39 | 0.25 (0.01;0.49) |
|  | Somewhat important | 0 | **0** | 0 | 0 | 0 |  |
|  | Quite important | 1 | 0 | **0** | 1 | 2 |  |
|  | Very important | 2 | 0 | 0 | **6** | 8 |  |
|  | Total | 27 | 0 | 3 | 19 | **49** |  |
|  | | | | | | | |
| Curing | Doesn’t apply/ not at all important | **0** | 0 | 0 | 2 | 2 | 0.09  (-0.15;0.32) |
|  | Somewhat important | 0 | **0** | 2 | 1 | 3 |  |
|  | Quite important | 0 | 1 | **5** | 6 | 12 |  |
|  | Very important | 1 | 2 | 2 | **28** | 33 |  |
|  | Total | 1 | 3 | 9 | 37 | **50** |  |
|  | | | | | | | |
| Alive | Doesn’t apply/ not at all important | **2** | 2 | 0 | 3 | 7 | 0.36 (0.03;0.70) |
|  | Somewhat important | 0 | **0** | 0 | 0 | 0 |  |
|  | Quite important | 0 | 1 | **4** | 3 | 8 |  |
|  | Very important | 3 | 0 | 5 | **27** | 35 |  |
|  | Total | 5 | 3 | 9 | 33 | **50** |  |
|  | | | | | | | |
| Enjoying life | Doesn’t apply/ not at all important | **4** | 2 | 6 | 4 | 16 | 0.33 (0.08;0.59) |
|  | Somewhat important | 1 | **1** | 0 | 1 | 3 |  |
|  | Quite important | 2 | 0 | **8** | 2 | 12 |  |
|  | Very important | 2 | 0 | 2 | **14** | 18 |  |
|  | Total | 9 | 3 | 16 | 21 | **49** |  |

| Item | Test  Retest | Doesn’t apply/ not at all important | Somewhat important | Quite important | Very important | Total | Weighted Kappa (95% CI) |
| --- | --- | --- | --- | --- | --- | --- | --- |
| Groceries | Doesn’t apply/ not at all important | **16** | 0 | 2 | 6 | 24 | 0.36 (0.10;0.62) |
|  | Somewhat important | 2 | **1** | 2 | 0 | 5 |  |
|  | Quite important | 1 | 0 | **5** | 5 | 11 |  |
|  | Very important | 3 | 0 | 3 | **4** | 10 |  |
|  | Total | 22 | 1 | 12 | 15 | **50** |  |
|  | | | | | | | |
| Wash and dress | Doesn’t apply/ not at all important | **23** | 1 | 2 | 7 | 33 | 0.44 (0.19;0.68) |
|  | Somewhat important | 0 | **0** | 0 | 0 | 0 |  |
|  | Quite important | 1 | 1 | **2** | 3 | 7 |  |
|  | Very important | 2 | 1 | 0 | **7** | 10 |  |
|  | Total | 26 | 3 | 4 | 17 | **50** |  |
|  | | | | | | | |
| Gardening | Doesn’t apply/ not at all important | **33** | 2 | 3 | 1 | 39 | 0.61 (0.37;0.85) |
|  | Somewhat important | 0 | **1** | 0 | 0 | 1 |  |
|  | Quite important | 2 | 0 | **4** | 1 | 7 |  |
|  | Very important | 0 | 0 | 3 | **0** | 3 |  |
|  | Total | 35 | 3 | 10 | 2 | **50** |  |
|  | | | | | | | |
| Sports | Doesn’t apply/ not at all important | **23** | 2 | 1 | 2 | 28 | 0.71 (0.51;0.92) |
|  | Somewhat important | 2 | **3** | 1 | 0 | 6 |  |
|  | Quite important | 1 | 2 | **6** | 2 | 11 |  |
|  | Very important | 0 | 0 | 1 | **4** | 5 |  |
|  | Total | 26 | 7 | 9 | 8 | **50** |  |
|  | | | | | | | |
| Hobbies | Doesn’t apply/ not at all important | **17** | 2 | 4 | 3 | 26 | 0.63 (0.43;0.83) |
|  | Somewhat important | 1 | **0** | 0 | 1 | 2 |  |
|  | Quite important | 1 | 0 | **4** | 0 | 5 |  |
|  | Very important | 1 | 0 | 4 | **12** | 17 |  |
|  | Total | 20 | 2 | 12 | 16 | **50** |  |

| Item | Test  Retest | Doesn’t apply/ not at all important | Somewhat important | Quite important | Very important | Total | Weighted Kappa (95% CI) |
| --- | --- | --- | --- | --- | --- | --- | --- |
| Driving | Doesn’t apply/ not at all important | **22** | 2 | 1 | 3 | 28 | 0.63 (0.42;0.83) |
|  | Somewhat important | 0 | **0** | 0 | 0 | 0 |  |
|  | Quite important | 4 | 0 | **2** | 3 | 9 |  |
|  | Very important | 1 | 1 | 2 | **9** | 13 |  |
|  | Total | 27 | 3 | 5 | 15 | **50** |  |
|  | | | | | | | |
| Outings | Doesn’t apply/ not at all important | **18** | 0 | 2 | 2 | 22 | 0.61 (0.39;0.82) |
|  | Somewhat important | 3 | **1** | 2 | 0 | 6 |  |
|  | Quite important | 1 | 2 | **3** | 3 | 9 |  |
|  | Very important | 2 | 1 | 2 | **8** | 13 |  |
|  | Total | 24 | 4 | 9 | 13 | **50** |  |
|  | | | | | | | |
| Visiting | Doesn’t apply/ not at all important | **16** | 1 | 4 | 5 | 26 | 0.38 (0.11;0.65) |
|  | Somewhat important | 2 | **3** | 2 | 0 | 7 |  |
|  | Quite important | 2 | 0 | **4** | 0 | 6 |  |
|  | Very important | 2 | 0 | 3 | **6** | 11 |  |
|  | Total | 22 | 4 | 13 | 11 | **50** |  |
|  | | | | | | | |
| Home | Doesn’t apply/ not at all important | **3** | 0 | 1 | 14 | 18 | 0.03  (-0.21;0.28) |
|  | Somewhat important | 0 | **0** | 0 | 0 | 0 |  |
|  | Quite important | 0 | 0 | **2** | 1 | 3 |  |
|  | Very important | 3 | 0 | 4 | **21** | 28 |  |
|  | Total | 6 | 0 | 7 | 36 | **49** |  |
|  | | | | | | | |
| Independence | Doesn’t apply/ not at all important | **2** | 0 | 5 | 11 | 18 | -0.01  (-0.24;0.22) |
|  | Somewhat important | 0 | **0** | 0 | 0 | 0 |  |
|  | Quite important | 1 | 0 | **1** | 5 | 7 |  |
|  | Very important | 5 | 0 | 1 | **19** | 25 |  |
|  | Total | 8 | 0 | 7 | 35 | **50** |  |

| Item | Test  Retest | Doesn’t apply/ not at all important | Somewhat important | Quite important | Very important | Total | Weighted Kappa (95% CI) |
| --- | --- | --- | --- | --- | --- | --- | --- |
| Extra | Doesn’t apply/ not at all important | **0** | 0 | 0 | 0 | 0 | nc |
|  | Somewhat important | 0 | **0** | 0 | 0 | 0 |  |
|  | Quite important | 0 | 0 | **0** | 0 | 0 |  |
|  | Very important | 0 | 0 | 0 | **2** | 2 |  |
|  | Total | 0 | 0 | 0 | 2 | **2** |  |

**Table 5. Crosstabulations of test-retest Baseline Status**

Retest

| Item | Test | Very bad | Bad | Mediocre | Satisfactory | Good | Very good | Total | Weighted Kappa (95% CI) |
| --- | --- | --- | --- | --- | --- | --- | --- | --- | --- |
| Better | Very bad | **5** | 2 | 0 | 0 | 0 | 0 | 7 | 0.70 (0.53;87) |
|  | Bad | 4 | **8** | 1 | 1 | 0 | 0 | 14 |  |
|  | Mediocre | 1 | 4 | **9** | 1 | 1 | 0 | 16 |  |
|  | Satisfactory | 0 | 1 | 0 | **0** | 1 | 0 | 2 |  |
|  | Good | 0 | 1 | 1 | 1 | **4** | 0 | 7 |  |
|  | Very good | 0 | 0 | 0 | 0 | 0 | **0** | 0 |  |
|  | Total | 10 | 16 | 11 | 3 | 6 | 0 | **46** |  |
|  | | | | | | | | | |
| Energy | Very bad | **2** | 3 | 2 | 1 | 0 | 0 | 8 | 0.38 (0.09;0.67) |
|  | Bad | 0 | **8** | 4 | 0 | 1 | 0 | 13 |  |
|  | Mediocre | 0 | 4 | **2** | 2 | 3 | 0 | 11 |  |
|  | Satisfactory | 2 | 0 | 0 | **0** | 1 | 0 | 3 |  |
|  | Good | 0 | 0 | 1 | 0 | **1** | 0 | 2 |  |
|  | Very good | 0 | 0 | 0 | 0 | 1 | **0** | 1 |  |
|  | Total | 4 | 15 | 9 | 3 | 7 | 0 | **38** |  |
|  | | | | | | | | | |
| Pain | Very bad | **2** | 1 | 2 | 0 | 0 | 0 | 5 | 0.71 (0.43;0.99) |
|  | Bad | 0 | **3** | 1 | 0 | 0 | 0 | 4 |  |
|  | Mediocre | 0 | 1 | **2** | 0 | 0 | 0 | 3 |  |
|  | Satisfactory | 0 | 0 | 0 | **0** | 0 | 0 | 0 |  |
|  | Good | 0 | 0 | 1 | 0 | **2** | 0 | 3 |  |
|  | Very good | 0 | 0 | 0 | 0 | 0 | **0** | 0 |  |
|  | Total | 2 | 5 | 6 | 0 | 2 | 0 | **15** |  |
|  | | | | | | | | | |
| Bowel movements | Very bad | **0** | 0 | 0 | 0 | 0 | 0 | 0 | nc |
|  | Bad | 0 | **0** | 1 | 0 | 0 | 0 | 1 |  |
|  | Mediocre | 0 | 0 | **0** | 0 | 1 | 0 | 1 |  |
|  | Satisfactory | 1 | 0 | 0 | **0** | 0 | 0 | 1 |  |
|  | Good | 0 | 0 | 0 | 0 | **1** | 0 | 1 |  |
|  | Very good | 0 | 0 | 0 | 0 | 0 | **0** | 0 |  |
|  | Total | 1 | 0 | 1 | 0 | 1 | 0 | **4** |  |
|  | | | | | | | | | |
| Shortness of breath | Very bad | **2** | 3 | 0 | 0 | 0 | 0 | 5 | 0.44 (0.17;0.71) |
|  | Bad | 2 | **9** | 2 | 0 | 0 | 0 | 13 |  |
|  | Mediocre | 1 | 0 | **4** | 2 | 0 | 0 | 7 |  |
|  | Satisfactory | 0 | 0 | 0 | **0** | 0 | 0 | 0 |  |
|  | Good | 0 | 1 | 1 | 0 | **0** | 0 | 2 |  |
|  | Very good | 0 | 0 | 0 | 0 | 0 | **0** | 0 |  |
|  | Total | 5 | 13 | 7 | 2 | 0 | 0 | **27** |  |

| Item | Test  Retest | Very bad | Bad | Mediocre | Satisfactory | Good | Very good | Total | Weighted Kappa (95% CI) |
| --- | --- | --- | --- | --- | --- | --- | --- | --- | --- |
| Walking | Very bad | **3** | 1 | 1 | 0 | 0 | 0 | 5 | 0.72 (0.53;0.92) |
|  | Bad | 2 | **8** | 5 | 0 | 0 | 0 | 15 |  |
|  | Mediocre | 0 | 2 | **5** | 0 | 3 | 0 | 10 |  |
|  | Satisfactory | 0 | 0 | 0 | **0** | 0 | 0 | 0 |  |
|  | Good | 0 | 0 | 0 | 0 | **0** | 0 | 0 |  |
|  | Very good | 0 | 0 | 0 | 0 | 1 | **1** | 2 |  |
|  | Total | 5 | 11 | 11 | 0 | 4 | 1 | **32** |  |
|  | | | | | | | | | |
| Appetite | Very bad | **0** | 1 | 0 | 0 | 0 | 0 | 1 | 0.80 (0.59;1.02) |
|  | Bad | 0 | **0** | 2 | 0 | 0 | 0 | 2 |  |
|  | Mediocre | 0 | 0 | **3** | 1 | 0 | 0 | 4 |  |
|  | Satisfactory | 0 | 0 | 1 | **0** | 0 | 0 | 1 |  |
|  | Good | 0 | 0 | 0 | 1 | **0** | 0 | 1 |  |
|  | Very good | 0 | 0 | 0 | 0 | 0 | **1** | 1 |  |
|  | Total | 0 | 1 | 6 | 2 | 0 | 1 | **10** |  |
|  | | | | | | | | | |
| Knowing what is wrong | Very bad | **0** | 2 | 0 | 0 | 0 | 0 | 2 | nc |
|  | Bad | 1 | **1** | 0 | 0 | 0 | 0 | 2 |  |
|  | Mediocre | 1 | 0 | **0** | 1 | 0 | 0 | 2 |  |
|  | Satisfactory | 0 | 0 | 0 | **0** | 0 | 0 | 0 |  |
|  | Good | 0 | 0 | 0 | 0 | **1** | 0 | 1 |  |
|  | Very good | 0 | 0 | 0 | 0 | 0 | **0** | 0 |  |
|  | Total | 2 | 4 | 0 | 1 | 1 | 0 | **7** |  |
|  | | | | | | | | | |
| Curing | Very bad | **1** | 1 | 2 | 0 | 0 | 0 | 4 | 0.29 (0;0.59) |
|  | Bad | 2 | **11** | 4 | 1 | 0 | 0 | 18 |  |
|  | Mediocre | 2 | 2 | **9** | 3 | 2 | 0 | 18 |  |
|  | Satisfactory | 0 | 0 | 1 | **0** | 1 | 0 | 2 |  |
|  | Good | 1 | 1 | 2 | 0 | **1** | 0 | 5 |  |
|  | Very good | 0 | 0 | 0 | 0 | 0 | **0** | 0 |  |
|  | Total | 6 | 15 | 18 | 4 | 4 | 0 | **47** |  |
|  | | | | | | | | | |
| Enjoying life | Very bad | **0** | 0 | 1 | 1 | 0 | 0 | 2 | 0.40 (0.14;0.67) |
|  | Bad | 0 | **0** | 2 | 1 | 3 | 0 | 6 |  |
|  | Mediocre | 1 | 2 | **6** | 0 | 0 | 0 | 9 |  |
|  | Satisfactory | 0 | 1 | 1 | **1** | 4 | 0 | 7 |  |
|  | Good | 0 | 0 | 0 | 0 | **1** | 1 | 2 |  |
|  | Very good | 0 | 0 | 0 | 0 | 2 | **1** | 3 |  |
|  | Total | 1 | 3 | 10 | 3 | 10 | 2 | **29** |  |
|  | | | | | | | | | |
| Groceries | Very bad | **1** | 1 | 0 | 0 | 0 | 0 | 2 | 0.57 (0.25;0.89) |
|  | Bad | 1 | **1** | 1 | 0 | 0 | 0 | 3 |  |
|  | Mediocre | 0 | 1 | **3** | 1 | 2 | 0 | 7 |  |
|  | Satisfactory | 0 | 0 | 0 | **0** | 0 | 0 | 0 |  |
|  | Good | 0 | 3 | 0 | 0 | **2** | 2 | 7 |  |
|  | Very good | 0 | 0 | 0 | 0 | 0 | **1** | 1 |  |
|  | Total | 2 | 6 | 4 | 1 | 4 | 3 | **20** |  |
|  | | | | | | | | | |
| Wash and dress | Very bad | **0** | 0 | 0 | 0 | 0 | 0 | 0 | 0.70 (0.52;0.88) |
|  | Bad | 0 | **0** | 1 | 1 | 0 | 0 | 2 |  |
|  | Mediocre | 1 | 1 | **1** | 1 | 0 | 0 | 4 |  |
|  | Satisfactory | 0 | 0 | 0 | **0** | 1 | 0 | 1 |  |
|  | Good | 0 | 0 | 1 | 0 | **2** | 2 | 5 |  |
|  | Very good | 0 | 0 | 0 | 0 | 0 | **2** | 2 |  |
|  | Total | 1 | 1 | 3 | 2 | 3 | 4 | **14** |  |

| Item | Test  Retest | Very bad | Bad | Mediocre | Satisfactory | Good | Very good | Total | Weighted Kappa (95% CI) |
| --- | --- | --- | --- | --- | --- | --- | --- | --- | --- |
| Gardening | Very bad | **0** | 0 | 0 | 0 | 1 | 0 | 1 | nc |
|  | Bad | 0 | **1** | 3 | 0 | 0 | 0 | 4 |  |
|  | Mediocre | 0 | 1 | **0** | 0 | 0 | 0 | 1 |  |
|  | Satisfactory | 0 | 1 | 0 | **0** | 0 | 0 | 1 |  |
|  | Good | 0 | 0 | 0 | 1 | **1** | 0 | 2 |  |
|  | Very good | 0 | 0 | 0 | 0 | 0 | **0** | 0 |  |
|  | Total | 0 | 3 | 3 | 1 | 2 | 0 | **9** |  |
|  | | | | | | | | | |
| Sports | Very bad | **2** | 1 | 0 | 1 | 0 | 0 | 4 | 0.64 (0.26;1.01) |
|  | Bad | 0 | **6** | 3 | 0 | 0 | 0 | 9 |  |
|  | Mediocre | 0 | 1 | **2** | 0 | 0 | 0 | 3 |  |
|  | Satisfactory | 0 | 0 | 0 | **1** | 0 | 0 | 1 |  |
|  | Good | 0 | 0 | 0 | 2 | **0** | 0 | 2 |  |
|  | Very good | 0 | 0 | 0 | 0 | 0 | **0** | 0 |  |
|  | Total | 2 | 8 | 5 | 4 | 0 | 0 | **19** |  |
|  | | | | | | | | | |
| Hobbies | Very bad | **1** | 0 | 0 | 0 | 1 | 0 | 2 | 0.36  (-0.2;0.74) |
|  | Bad | 0 | **1** | 1 | 0 | 0 | 0 | 2 |  |
|  | Mediocre | 1 | 1 | **2** | 0 | 2 | 1 | 7 |  |
|  | Satisfactory | 0 | 0 | 0 | **0** | 1 | 0 | 1 |  |
|  | Good | 0 | 0 | 2 | 1 | **3** | 2 | 8 |  |
|  | Very good | 0 | 0 | 1 | 0 | 0 | **0** | 1 |  |
|  | Total | 2 | 2 | 6 | 1 | 7 | 3 | **21** |  |
|  | | | | | | | | | |
| Driving | Very bad | **2** | 0 | 0 | 0 | 1 | 0 | 3 | 0.75 (0.44;1.06) |
|  | Bad | 0 | **0** | 1 | 0 | 0 | 0 | 1 |  |
|  | Mediocre | 0 | 0 | **0** | 0 | 0 | 0 | 0 |  |
|  | Satisfactory | 0 | 0 | 0 | **1** | 0 | 0 | 1 |  |
|  | Good | 0 | 0 | 1 | 0 | **4** | 3 | 8 |  |
|  | Very good | 0 | 0 | 0 | 0 | 1 | **3** | 4 |  |
|  | Total | 2 | 0 | 2 | 1 | 6 | 6 | **17** |  |
|  | | | | | | | | | |
| Outings | Very bad | **0** | 0 | 0 | 0 | 1 | 0 | 1 | 0.54 (0.19;0.89) |
|  | Bad | 1 | **3** | 2 | 1 | 0 | 0 | 7 |  |
|  | Mediocre | 1 | 2 | **3** | 0 | 0 | 0 | 6 |  |
|  | Satisfactory | 0 | 0 | 0 | **0** | 1 | 0 | 1 |  |
|  | Good | 0 | 1 | 1 | 0 | **2** | 1 | 5 |  |
|  | Very good | 0 | 0 | 0 | 0 | 1 | **1** | 2 |  |
|  | Total | 2 | 6 | 6 | 1 | 5 | 2 | **22** |  |
|  | | | | | | | | | |
| Visiting | Very bad | **0** | 0 | 0 | 0 | 2 | 0 | 2 | 0.25 (-0.25;0.76) |
|  | Bad | 1 | **1** | 0 | 0 | 0 | 0 | 2 |  |
|  | Mediocre | 0 | 0 | **5** | 0 | 0 | 0 | 5 |  |
|  | Satisfactory | 0 | 1 | 0 | **0** | 0 | 0 | 1 |  |
|  | Good | 0 | 0 | 1 | 1 | **5** | 0 | 7 |  |
|  | Very good | 0 | 0 | 1 | 0 | 0 | **0** | 1 |  |
|  | Total | 1 | 2 | 7 | 1 | 7 | 0 | **18** |  |
|  | | | | | | | | | |
| Home | Very bad | **0** | 1 | 0 | 0 | 1 | 0 | 2 | 0.37  (-0.2;0.75) |
|  | Bad | 0 | **0** | 0 | 0 | 1 | 0 | 1 |  |
|  | Mediocre | 0 | 0 | **0** | 0 | 1 | 0 | 1 |  |
|  | Satisfactory | 0 | 0 | 0 | **0** | 2 | 0 | 2 |  |
|  | Good | 1 | 0 | 0 | 2 | **9** | 2 | 14 |  |
|  | Very good | 0 | 0 | 0 | 1 | 1 | **6** | 8 |  |
|  | Total | 1 | 1 | 0 | 3 | 15 | 8 | **28** |  |

| Item | Test  Retest | Very bad | Bad | Mediocre | Satisfactory | Good | Very good | Total | Weighted Kappa (95% CI) |
| --- | --- | --- | --- | --- | --- | --- | --- | --- | --- |
| Indepen-dence | Very bad | **0** | 0 | 0 | 1 | 1 | 0 | 2 | 0.59 (0.34;0.84) |
|  | Bad | 1 | **2** | 1 | 1 | 0 | 0 | 5 |  |
|  | Mediocre | 0 | 0 | **3** | 0 | 1 | 0 | 4 |  |
|  | Satisfactory | 0 | 1 | 0 | **0** | 0 | 1 | 2 |  |
|  | Good | 0 | 0 | 0 | 3 | **3** | 3 | 9 |  |
|  | Very good | 0 | 0 | 0 | 0 | 2 | **2** | 4 |  |
|  | Total | 1 | 3 | 4 | 5 | 7 | 6 | **26** |  |
|  | | | | | | | | | |
| Extra | Very bad | **0** | 1 | 0 | 0 | 0 | 0 | 1 | nc |
|  | Bad | 0 | **0** | 0 | 0 | 0 | 0 | 0 |  |
|  | Mediocre | 0 | 0 | **0** | 0 | 0 | 0 | 0 |  |
|  | Satisfactory | 0 | 0 | 0 | **0** | 0 | 0 | 0 |  |
|  | Good | 0 | 0 | 0 | 0 | **0** | 0 | 0 |  |
|  | Very good | 0 | 0 | 0 | 0 | 0 | **1** | 1 |  |
|  | Total | 0 | 0 | 0 | 0 | 0 | 1 | **2** |  |

**Table 6. Crosstabulations of test-retest baseline prevention/preservation or improvement**

Retest

| Item | Test | Prevention/  Preservation | Improvement | Total | Kappa |
| --- | --- | --- | --- | --- | --- |
| Better | Good | 2 | 5 | 7 | 0.28  (-0.10;0.67) |
|  | Better | 2 | 37 | 39 |  |
|  | Total | 4 | 42 | 46 |  |
|  | | | | |  |
| Energy | Preservation | 4 | 3 | 7 | 0.54 (0.18;0.89) |
|  | Improvement | 2 | 29 | 31 |  |
|  | Total | 6 | 32 | 38 |  |
|  | | | | |  |
| Pain | Prevention | 1 | 1 | 2 | 0.63  (-0,1;1.23) |
|  | Improvement | 0 | 13 | 13 |  |
|  | Total | 1 | 14 | 15 |  |
|  | | | | |  |
| Bowel movements | Prevention | 0 | 0 | 0 | nc |
|  | Improvement | 1 | 3 | 4 |  |
|  | Total | 1 | 3 | 4 |  |
|  | | | | |  |
| Shortness of breath | Prevention | 0 | 1 | 1 | nc |
|  | Improvement | 0 | 26 | 26 |  |
|  | Total | 0 | 27 | 27 |  |
|  | | | | |  |
| Walking | Preservation | 2 | 1 | 3 | 0.52 (0.05;0.99) |
|  | Improvement | 2 | 27 | 29 |  |
|  | Total | 4 | 28 | 32 |  |
|  | | | | | |
| Appetite | Preservation | 2 | 2 | 4 | 0.35 (-0.24;0.94) |
|  | Improvement | 1 | 5 | 6 |  |
|  | Total | 3 | 7 | 10 |  |
|  | | | | | |
| Curing | Controlling | 5 | 3 | 8 | 0.49 (0.17;0.82) |
|  | Curing | 4 | 33 | 37 |  |
|  | Total | 9 | 36 | 45 |  |
|  | | | | | |
| Enjoy | Preservation | 10 | 3 | 13 | 0.65 (0.37;0.93) |
|  | Improvement | 2 | 14 | 16 |  |
|  | Total | 12 | 17 | 29 |  |
|  | | | | | |
| Groceries | Preservation | 6 | 4 | 10 | 0.40 (0.01;0.79) |
|  | Improvement | 2 | 8 | 10 |  |
|  | Total | 8 | 12 | 20 |  |
| Item | Test  Retest | Prevention/  Preservation | Improvement | Total | Kappa |
| Wash and dress | Preservation | 5 | 2 | 7 | 0.14  (-0.35;0.64) |
|  | Improvement | 4 | 3 | 7 |  |
|  | Total | 9 | 5 | 14 |  |
|  | | | | | |
| Garden | Preservation | 3 | 0 | 3 | nc |
|  | Improvement | 1 | 5 | 6 |  |
|  | Total | 4 | 5 | 9 |  |
|  | | | | | |
| Sports | Preservation | 3 | 0 | 3 | 0.68 (0.29;1) |
|  | Improvement | 2 | 13 | 15 |  |
|  | Total | 5 | 13 | 18 |  |
|  | | | | | |
| Hobbies | Preservation | 9 | 2 | 11 | 0.27  (-0.14;0.68) |
|  | Improvement | 5 | 4 | 9 |  |
|  | Total | 14 | 6 | 20 |  |
|  | | | | | |
| Driving | Preservation | 10 | 1 | 11 | 0.27  (-0.18;0.73) |
|  | Improvement | 4 | 2 | 6 |  |
|  | Total | 14 | 3 | 17 |  |
|  | | | | | |
| Outings | Preservation | 3 | 4 | 7 | 0.16  (-0.27;0.59) |
|  | Improvement | 4 | 11 | 15 |  |
|  | Total | 7 | 15 | 22 |  |
|  | | | | | |
| Visiting | Preservation | 5 | 4 | 9 | 0.22  (-0.23;0.67) |
|  | Improvement | 3 | 6 | 9 |  |
|  | Total | 8 | 10 | 18 |  |
|  | | | | | |
| Home | Preservation | 23 | 3 | 26 | 0.26 (0;0.77) |
|  | Improvement | 1 | 1 | 2 |  |
|  | Total | 24 | 4 | 28 |  |
|  | | | | | |
| Independence | Preservation | 10 | 6 | 16 | 0.31  (-0.05;0.66) |
|  | Improvement | 3 | 7 | 10 |  |
|  | Total | 13 | 13 | 26 |  |
|  | | | | | |
| Extra | Preservation | 1 | 0 | 1 | nc |
|  | Improvement | 0 | 1 | 1 |  |
|  | Total | 1 | 1 | 2 |  |

**References**

(1) De Vet HCW, Terwee CB, Mokkink LB, Knol DL. Measurement in Medicine. A Practical Guide. 1st ed. Cambridge: Cambridge University Press; 2011.

(2) Sim J, Wright CC. The kappa statistic in reliability studies: use, interpretation, and sample size requirements. Phys Ther 2005 Mar;85(3):257-268.

(3) Lowry R. VassarStats: Website for statistical computation. 1998-2021.

(4) Landis JR, Koch GG. The measurement of observer agreement for categorical data. Biometris 1977;33(1):159-174.
