# Supplementary material for: Adaptation of the Patient Benefit Assessment Scale for Hospitalised Older Patients: development, reliability and validity of the P-BAS picture version
Source: BMC Geriatr. 2022 Jan 11;22:43. doi: 10.1186/s12877-021-02708-7 (PMC8751090; doi:10.1186/s12877-021-02708-7)
Supplement: Supplementary file 6 — Additional file 6. Test-retest Follow-up on item level. [file 12877_2021_2708_MOESM6_ESM.docx]

**Additional file 6. Test-retest Follow-up on item level**

**Adaptation of the Patient Benefit Assessment Scale for Hospitalised Older Patients: development, reliability and validity of the P-BAS Picture version**

**Authors:**

1. Maria Johanna van der Kluit, MSc RN (Corresponding author)

University of Groningen, University Medical Center Groningen, University Center for Geriatric Medicine, Hanzeplein 1, 9700 RB Groningen, The Netherlands

[m.j.van.der.kluit@umcg.nl](mailto:m.j.van.der.kluit@umcg.nl)

+31503613921

1. Geke J. Dijkstra, PhD

University of Groningen, University Medical Center Groningen, Department of Health Sciences, Applied Health Research, Groningen, The Netherlands

NHL Stenden University of Applied Sciences, Research Group Living, Wellbeing and Care for Older People, Leeuwarden, The Netherlands

[g.j.dijkstra@umcg.nl](mailto:g.j.dijkstra@umcg.nl)

1. Sophia E. de Rooij, MD PhD

University of Groningen, University Medical Center Groningen, University Center for Geriatric Medicine, Groningen, The Netherlands

[sejaderooij@gmail.com](mailto:sejaderooij@gmail.com)

**Additional file 6. Test-retest Follow-up on item level**

Percentage of agreement, Cohen’s kappa with quadratic weighting and maximum attainable kappa (1,2) were calculated per item for agreement on status at follow-up. Kappa values and confidence intervals were calculated using IBM SPSS 27. Maximal attainable kappa values were calculated using online calculator (3). For the interpretation of the kappa values, the classification of Landis and Koch (4) was used.

Table 1 shows the weighted kappa values for follow-up status items in descending order. Four items had almost perfect agreement, seven items had substantial agreement, five moderate agreement, two fair agreement, one slight agreement, and the item ‘wash and dress’, had an agreement smaller then chance. When the weighted kappa was calculated as a proportion of the maximum attainable kappa, eight items had almost perfect agreement, six items had substantial agreement, two items moderate agreement, two fair agreement, and one no agreement. The mean of all the weighted kappa values showed moderate agreement, when calculated as a proportion of the maximum attainable kappa, substantial agreement. Full crosstabulations of all items are shown in Table 2.

Table 1. Cohen’s weighted kappa with quadratic weighting for follow-up status items in descending order (n=5-48)

| Item | n | % agreement | Weighted Kappa (95% CI) | K_max_ | Weighted K/ K_max_ |
| --- | --- | --- | --- | --- | --- |
| Driving | 31 | 70.97 | 0.90 (0.80;0.99) | 0.98 | 0.91 |
| Appetite | 12 | 66.67 | 0.88 (0.74;1.02) | nc | nc |
| Bowel movements | 11 | 63.64 | 0.82 (0.68;0.96) | 0.87 | 0.94 |
| Pain | 18 | 50.00 | 0.81 (0.70;0.92) | 0.89 | 0.90 |
| Better | 48 | 70.83 | 0.79 (0.62;0.96) | 0.87 | 0.91 |
| Walking | 30 | 50.00 | 0.76 (0.60;0.91) | 0.89 | 0.63 |
| Groceries | 23 | 56.52 | 0.74 (0.48;1.00)) | 1 | 0.74 |
| Curing | 47 | 53.19 | 0.74 (0.60;0.88) | 0.89 | 0.83 |
| Enjoy | 38 | 52.63 | 0.67 (0.45;0.89) | 0.83 | 0.81 |
| Shortness of breath | 21 | 33.33 | 0.66 (0.45;0.86) | 0.75 | 0.88 |
| Garden | 24 | 50.00 | 0.66 (0.43;0.90) | 0.92 | 0.72 |
| Hobbies | 21 | 47.62 | 0.60 (0.30;0.90) | 0.65 | 0.91 |
| Energy | 39 | 43.59 | 0.54 (0.29;0.80) | 0.96 | 0.57 |
| Knowing what is wrong | 22 | 45.45 | 0.51 (0.25;0.77) | 0.71 | 0.72 |
| Sports | 15 | 46.67 | 0.48 (-0.03;0.99) | nc | nc |
| Independence | 38 | 55.26 | 0.44 (0.15;0.73) | 0.96 | 0.47 |
| Outings | 17 | 52.94 | 0.39 (0.07;0.72) | 0.53 | 0.73 |
| Visiting | 17 | 70.59 | 0.26 (-0.12;0.63) | 0.80 | 0.32 |
| Home | 36 | 75.00 | 0.18 (-0.32;0.67) | 0.76 | 0.23 |
| Wash and dress | 18 | 33.33 | 0.15 (-0.12;0.43) | 0.24 | 0.63 |
| Extra | 5 | 80.00 | nc | nc | nc |
| Mean | 25.29 | 55.63 | 0.60 | 0.82 | 0.71 |

K= kappa K_max_=maximum attainable kappa CI= Confidence interval nc= not calculated

Table 2. Crosstabulations of test-retest Follow-up status

Retest

| Item | Test | Very bad | Bad | Mediocre | Satisfactory | Good | Very good | Total | Weighted Kappa (95% CI) |
| --- | --- | --- | --- | --- | --- | --- | --- | --- | --- |
| Better | Very bad | **0** | 0 | 0 | 0 | 0 | 0 | 0 | 0.79 (0.62;0.96) |
|  | Bad | 1 | **0** | 0 | 0 | 1 | 0 | 2 |  |
|  | Mediocre | 0 | 0 | **8** | 1 | 0 | 0 | 9 |  |
|  | Satisfactory | 0 | 0 | 1 | **5** | 5 | 0 | 11 |  |
|  | Good | 0 | 0 | 0 | 1 | **18** | 2 | 21 |  |
|  | Very good | 0 | 0 | 0 | 0 | 2 | **3** | 5 |  |
|  | Total | 1 | 0 | 9 | 7 | 26 | 5 | **48** |  |
|  | | | | | | | | | |
| Energy | Very bad | **0** | 0 | 0 | 0 | 0 | 0 | 0 | 0.54 (0.29;0.80) |
|  | Bad | 0 | **1** | 0 | 0 | 1 | 0 | 2 |  |
|  | Mediocre | 0 | 1 | **7** | 2 | 0 | 0 | 10 |  |
|  | Satisfactory | 0 | 0 | 1 | **3** | 7 | 0 | 11 |  |
|  | Good | 0 | 0 | 1 | 7 | **6** | 2 | 16 |  |
|  | Very good | 0 | 0 | 0 | 0 | 0 | **0** | 0 |  |
|  | Total | 0 | 2 | 9 | 12 | 14 | 2 | **39** |  |
|  | | | | | | | | | |
| Pain | Very bad | **0** | 0 | 0 | 0 | 0 | 0 | 0 | 0.81 (0.70;0.92) |
|  | Bad | 0 | **0** | 0 | 0 | 0 | 0 | 0 |  |
|  | Mediocre | 0 | 1 | **4** | 1 | 0 | 0 | 6 |  |
|  | Satisfactory | 0 | 0 | 0 | **0** | 2 | 0 | 2 |  |
|  | Good | 0 | 0 | 0 | 1 | **4** | 2 | 7 |  |
|  | Very good | 0 | 0 | 0 | 0 | 2 | **1** | 3 |  |
|  | Total | 0 | 1 | 4 | 2 | 8 | 3 | **18** |  |
|  | | | | | | | | | |
| Bowel movements | Very bad | **0** | 0 | 1 | 0 | 0 | 0 | 1 | 0.82 (0.68;0.96) |
|  | Bad | 0 | **1** | 0 | 0 | 0 | 0 | 1 |  |
|  | Mediocre | 0 | 0 | **1** | 0 | 0 | 0 | 1 |  |
|  | Satisfactory | 0 | 0 | 1 | **1** | 0 | 0 | 2 |  |
|  | Good | 0 | 0 | 0 | 0 | **4** | 1 | 5 |  |
|  | Very good | 0 | 0 | 0 | 0 | 1 | **0** | 1 |  |
|  | Total | 0 | 1 | 3 | 1 | 5 | 1 | **11** |  |
|  | | | | | | | | | |
| Shortness of breath | Very bad | **0** | 0 | 0 | 0 | 0 | 0 | 0 | 0.66 (0.45;0.86) |
|  | Bad | 0 | **1** | 0 | 0 | 0 | 0 | 1 |  |
|  | Mediocre | 0 | 2 | **3** | 0 | 1 | 0 | 6 |  |
|  | Satisfactory | 0 | 0 | 3 | **1** | 0 | 0 | 4 |  |
|  | Good | 0 | 0 | 2 | 0 | **2** | 5 | 9 |  |
|  | Very good | 0 | 0 | 0 | 0 | 1 | **0** | 1 |  |
|  | Total | 0 | 3 | 8 | 1 | 4 | 5 | **21** |  |
|  | | | | | | | | | |
| Walking | Very bad | **1** | 0 | 0 | 0 | 0 | 0 | 1 | 0.76 (0.60;0.91) |
|  | Bad | 0 | **0** | 1 | 0 | 0 | 0 | 1 |  |
|  | Mediocre | 0 | 1 | **5** | 1 | 0 | 0 | 7 |  |
|  | Satisfactory | 0 | 0 | 4 | **3** | 3 | 0 | 10 |  |
|  | Good | 0 | 0 | 0 | 2 | **6** | 1 | 9 |  |
|  | Very good | 0 | 0 | 0 | 1 | 1 | **0** | 2 |  |
|  | Total | 1 | 1 | 10 | 7 | 10 | 1 | **30** |  |
|  | | | | | | | | | |
| Appetite | Very bad | **0** | 0 | 0 | 0 | 0 | 0 | 0 | 0.88 (0.74;1.02) |
|  | Bad | 1 | **0** | 0 | 0 | 0 | 0 | 1 |  |
|  | Mediocre | 0 | 0 | **0** | 0 | 0 | 0 | 0 |  |
|  | Satisfactory | 0 | 0 | 0 | **0** | 2 | 0 | 2 |  |
|  | Good | 0 | 0 | 0 | 0 | **5** | 1 | 6 |  |
|  | Very good | 0 | 0 | 0 | 0 | 0 | **3** | 3 |  |
|  | Total | 1 | 0 | 0 | 0 | 7 | 4 | **12** |  |

| Item | Test  Retest | Very bad | | Bad | Mediocre | Satisfactory | Good | Very good | Total | Weighted Kappa (95% CI) |
| --- | --- | --- | --- | --- | --- | --- | --- | --- | --- | --- |
| Knowing what is wrong | Very bad | **0** | | 0 | 0 | 0 | 0 | 0 | 0 | 0.51 (0.25;0.77) |
|  | Bad | 0 | | **0** | 2 | 0 | 1 | 0 | 3 |  |
|  | Mediocre | 0 | | 0 | **0** | 0 | 1 | 0 | 1 |  |
|  | Satisfactory | 1 | | 0 | 0 | **0** | 1 | 1 | 3 |  |
|  | Good | 0 | | 0 | 0 | 0 | **7** | 3 | 10 |  |
|  | Very good | 0 | | 0 | 0 | 0 | 2 | **3** | 5 |  |
|  | Total | 1 | | 0 | 2 | 0 | 12 | 7 | **22** |  |
|  | | | | | | | | | | |
| Curing | Very bad | | **0** | 0 | 0 | 0 | 0 | 0 | 0 | 0.74 (0.60;0.88) |
|  | Bad | | 0 | **1** | 0 | 0 | 0 | 0 | 1 |  |
|  | Mediocre | | 0 | 2 | **5** | 0 | 2 | 0 | 9 |  |
|  | Satisfactory | | 0 | 0 | 3 | **1** | 7 | 0 | 11 |  |
|  | Good | | 0 | 0 | 0 | 1 | **12** | 3 | 16 |  |
|  | Very good | | 0 | 0 | 0 | 1 | 3 | **6** | 10 |  |
|  | Total | | 0 | 3 | 8 | 3 | 24 | 9 | **47** |  |
|  | | | | | | | | | | |
| Enjoying life | Very bad | | **0** | 0 | 0 | 0 | 0 | 0 | 0 | 0.67 (0.45;0.89) |
|  | Bad | | 0 | **0** | 0 | 0 | 0 | 0 | 0 |  |
|  | Mediocre | | 0 | 2 | **3** | 3 | 0 | 1 | 9 |  |
|  | Satisfactory | | 0 | 0 | 0 | **1** | 5 | 0 | 6 |  |
|  | Good | | 0 | 0 | 1 | 1 | **11** | 2 | 15 |  |
|  | Very good | | 0 | 0 | 0 | 0 | 3 | **5** | 8 |  |
|  | Total | | 0 | 2 | 4 | 5 | 19 | 8 | **38** |  |
|  | | | | | | | | | | |
| Groceries | Very bad | | **0** | 0 | 0 | 0 | 0 | 0 | 0 | 0.74 (0.48;1.00) |
|  | Bad | | 0 | **1** | 0 | 0 | 0 | 0 | 1 |  |
|  | Mediocre | | 0 | 0 | **1** | 0 | 0 | 0 | 1 |  |
|  | Satisfactory | | 0 | 0 | 0 | **1** | 2 | 0 | 3 |  |
|  | Good | | 0 | 0 | 0 | 2 | **9** | 3 | 14 |  |
|  | Very good | | 0 | 0 | 0 | 0 | 3 | **1** | 4 |  |
|  | Total | | 0 | 1 | 1 | 3 | 14 | 4 | **23** |  |
|  | | | | | | | | | | |
| Wash and dress | Very bad | | **0** | 0 | 0 | 0 | 0 | 0 | 0 | 0.15  (-.12;0.43) |
|  | Bad | | 0 | **0** | 0 | 0 | 0 | 0 | 0 |  |
|  | Mediocre | | 0 | 0 | **0** | 0 | 0 | 0 | 0 |  |
|  | Satisfactory | | 0 | 0 | 1 | **0** | 2 | 0 | 3 |  |
|  | Good | | 0 | 1 | 0 | 2 | **6** | 4 | 13 |  |
|  | Very good | | 0 | 0 | 0 | 0 | 2 | **0** | 2 |  |
|  | Total | | 0 | 1 | 1 | 2 | 10 | 4 | **18** |  |
|  | | | | | | | | | | |
| Gardening | Very bad | | **0** | 0 | 1 | 0 | 0 | 0 | 1 | 0.66 (0.43;0.90) |
|  | Bad | | 0 | **1** | 1 | 0 | 0 | 0 | 2 |  |
|  | Mediocre | | 0 | 1 | **3** | 1 | 0 | 0 | 5 |  |
|  | Satisfactory | | 0 | 0 | 0 | **1** | 3 | 1 | 5 |  |
|  | Good | | 0 | 1 | 0 | 1 | **6** | 1 | 9 |  |
|  | Very good | | 0 | 0 | 0 | 0 | 1 | **1** | 2 |  |
|  | Total | | 0 | 3 | 5 | 3 | 10 | 3 | **24** |  |
|  | | | | | | | | | | |
| Sports | Very bad | | **0** | 0 | 0 | 0 | 0 | 0 | 0 | 0.48  (-.03;0.99) |
|  | Bad | | 0 | **1** | 1 | 0 | 0 | 1 | 3 |  |
|  | Mediocre | | 0 | 0 | **2** | 1 | 1 | 0 | 4 |  |
|  | Satisfactory | | 0 | 0 | 1 | **1** | 1 | 0 | 3 |  |
|  | Good | | 0 | 0 | 0 | 1 | **2** | 1 | 4 |  |
|  | Very good | | 0 | 0 | 0 | 0 | 0 | **1** | 1 |  |
|  | Total | | 0 | 1 | 4 | 3 | 4 | 3 | **15** |  |

| Item | Test  Retest | Very bad | Bad | Mediocre | Satisfactory | Good | Very good | Total | Weighted Kappa (95% CI) | |
| --- | --- | --- | --- | --- | --- | --- | --- | --- | --- | --- |
| Hobbies | Very bad | **0** | 0 | 0 | 0 | 0 | 0 | 0 | 0.60 (0.30;0.90) | |
|  | Bad | 1 | **0** | 0 | 0 | 0 | 0 | 1 |  |  |
|  | Mediocre | 0 | 1 | **3** | 0 | 0 | 0 | 4 |  |  |
|  | Satisfactory | 0 | 0 | 0 | **0** | 2 | 0 | 2 |  |  |
|  | Good | 0 | 1 | 3 | 0 | **6** | 3 | 13 |  |  |
|  | Very good | 0 | 0 | 0 | 0 | 0 | **1** | 1 |  |  |
|  | Total | 1 | 2 | 6 | 0 | 8 | 4 | **21** |  |  |
|  | | | | | | | | | | |
| Driving | Very bad | **1** | 1 | 0 | 0 | 0 | 0 | 2 | 0.90 (0.80;0.99) | |
|  | Bad | 1 | **1** | 0 | 0 | 0 | 0 | 2 |  |  |
|  | Mediocre | 0 | 0 | **0** | 1 | 0 | 0 | 1 |  |  |
|  | Satisfactory | 0 | 0 | 1 | **1** | 1 | 0 | 3 |  |  |
|  | Good | 0 | 0 | 1 | 0 | **15** | 2 | 18 |  |  |
|  | Very good | 0 | 0 | 0 | 0 | 1 | **4** | 5 |  |  |
|  | Total | 2 | 2 | 2 | 2 | 17 | 6 | **31** |  |  |
|  | | | | | | | | | | |
| Outings | Very bad | **0** | 0 | 0 | 0 | 0 | 0 | 0 | 0.39 (0.07;0.72) | |
|  | Bad | 0 | **0** | 0 | 0 | 0 | 0 | 0 |  |  |
|  | Mediocre | 0 | 0 | **1** | 1 | 3 | 0 | 5 |  |  |
|  | Satisfactory | 0 | 0 | 1 | **1** | 1 | 0 | 3 |  |  |
|  | Good | 0 | 0 | 0 | 0 | **7** | 1 | 8 |  |  |
|  | Very good | 0 | 0 | 0 | 0 | 1 | **0** | 1 |  |  |
|  | Total | 0 | 0 | 2 | 2 | 12 | 1 | **17** |  |  |
|  | | | | | | | | | | |
| Visiting | Very bad | **0** | 0 | 0 | 0 | 0 | 0 | 0 | | 0.26  (-.12;0.63) |
|  | Bad | 0 | **0** | 0 | 0 | 0 | 0 | 0 | |  |
|  | Mediocre | 0 | 0 | **0** | 0 | 1 | 0 | 1 | |  |
|  | Satisfactory | 0 | 0 | 0 | **1** | 0 | 0 | 1 | |  |
|  | Good | 0 | 0 | 1 | 1 | **10** | 2 | 14 | |  |
|  | Very good | 0 | 0 | 0 | 0 | 0 | **1** | 1 | |  |
|  | Total | 0 | 0 | 1 | 2 | 11 | 3 | **17** | |  |
|  | | | | | | | | | | |
| Home | Very bad | **0** | 0 | 0 | 0 | 0 | 0 | 0 | | 0.18  (-.32;0.67) |
|  | Bad | 0 | **0** | 0 | 0 | 0 | 0 | 0 | |  |
|  | Mediocre | 0 | 0 | **0** | 0 | 0 | 0 | 0 | |  |
|  | Satisfactory | 0 | 0 | 0 | **0** | 1 | 0 | 1 | |  |
|  | Good | 0 | 0 | 0 | 1 | **22** | 4 | 27 | |  |
|  | Very good | 0 | 0 | 1 | 0 | 2 | **5** | 8 | |  |
|  | Total | 0 | 0 | 1 | 1 | 25 | 9 | **36** | |  |
|  | | | | | | | | | | |
| Independence | Very bad | **0** | 0 | 0 | 0 | 0 | 0 | 0 | 0.44 (0.15;0.73) | |
|  | Bad | 0 | **0** | 0 | 0 | 0 | 0 | 0 |  |  |
|  | Mediocre | 0 | 0 | **1** | 0 | 1 | 0 | 2 |  |  |
|  | Satisfactory | 0 | 0 | 0 | **1** | 1 | 0 | 2 |  |  |
|  | Good | 0 | 0 | 1 | 4 | **15** | 5 | 25 |  |  |
|  | Very good | 0 | 0 | 0 | 0 | 5 | **4** | 9 |  |  |
|  | Total | 0 | 0 | 2 | 5 | 22 | 9 | **38** |  |  |
|  | | | | | | | | | | |
| Extra | Very bad | **1** | 0 | 0 | 0 | 0 | 0 | 1 | nc | |
|  | Bad | 0 | **0** | 0 | 0 | 0 | 0 | 0 |  |  |
|  | Mediocre | 0 | 0 | **0** | 0 | 0 | 0 | 0 |  |  |
|  | Satisfactory | 0 | 0 | 0 | **0** | 0 | 0 | 0 |  |  |
|  | Good | 0 | 0 | 0 | 0 | **3** | 1 | 4 |  |  |
|  | Very good | 0 | 0 | 0 | 0 | 0 | **0** | 0 |  |  |
|  | Total | 1 | 0 | 0 | 0 | 3 | 1 | **5** |  |  |

**References**

(1) De Vet HCW, Terwee CB, Mokkink LB, Knol DL. Measurement in Medicine. A Practical Guide. 1st ed. Cambridge: Cambridge University Press; 2011.

(2) Sim J, Wright CC. The kappa statistic in reliability studies: use, interpretation, and sample size requirements. Phys Ther 2005 Mar;85(3):257-268.

(3) Lowry R. VassarStats: Website for statistical computation. 1998-2021.

(4) Landis JR, Koch GG. The measurement of observer agreement for categorical data. Biometris 1977;33(1):159-174.
