# Supplementary material for: Adaptation of the Patient Benefit Assessment Scale for Hospitalised Older Patients: development, reliability and validity of the P-BAS picture version
Source: BMC Geriatr. 2022 Jan 11;22:43. doi: 10.1186/s12877-021-02708-7 (PMC8751090; doi:10.1186/s12877-021-02708-7)
Supplement: Supplementary file 7 — Additional file 7. Crosstabulations validity hypotheses baseline importance of goals. [file 12877_2021_2708_MOESM7_ESM.docx]

**Additional file 7. Crosstabulations validity hypotheses baseline importance of goals**

**Adaptation of the Patient Benefit Assessment Scale for Hospitalised Older Patients: development, reliability and validity of the P-BAS Picture version**

**Authors:**

1. Maria Johanna van der Kluit, MSc RN (Corresponding author)

University of Groningen, University Medical Center Groningen, University Center for Geriatric Medicine, Hanzeplein 1, 9700 RB Groningen, The Netherlands

[m.j.van.der.kluit@umcg.nl](mailto:m.j.van.der.kluit@umcg.nl)

+31503613921

1. Geke J. Dijkstra, PhD

University of Groningen, University Medical Center Groningen, Department of Health Sciences, Applied Health Research, Groningen, The Netherlands

NHL Stenden University of Applied Sciences, Research Group Living, Wellbeing and Care for Older People, Leeuwarden, The Netherlands

[g.j.dijkstra@umcg.nl](mailto:g.j.dijkstra@umcg.nl)

1. Sophia E. de Rooij, MD PhD

University of Groningen, University Medical Center Groningen, University Center for Geriatric Medicine, Groningen, The Netherlands

[sejaderooij@gmail.com](mailto:sejaderooij@gmail.com)

**Additional file 7. Crosstabulations validity hypotheses baseline importance of goals**

| Hypothesis | Answer P-BAS-P | Answer VMS and/or RSCL | | Total | Cramér’s V |
| --- | --- | --- | --- | --- | --- |
|  |  | n (% within VMS and/or RSCL) | | |  |
|  |  | no | yes |  |  |
| Participants who indicated a lack of appetite on the VMS and/or the RSCL, are expected to have a higher importance on the goal ‘improving appetite’.* | Doesn’t apply/  not at all important | 105 (95) | 37 (64) | 142 (84) | .41 |
|  | Somewhat important | 2 (2) | 2 (3) | 4 (2) |  |
|  | Quite important | 3 (3) | 11 (19) | 14 (8) |  |
|  | Very important | 1 (1) | 8 (14) | 9 (5) |  |
|  | Total | 111 | 58 | 169 |  |
|  | | | | | |
|  | Answer P-BAS-P | Answer RSCL | | Total | Cramér’s V |
|  |  | n (% within RSCL) | | |  |
|  |  | no | yes |  |  |
| Participants who indicated tiredness and/ or lack of energy on the RSCL, are expected to have a higher importance on the goal ‘improving energy’.* | Doesn’t apply/  not at all important | 24 (47) | 24 (20) | 48 (28) | .26 |
|  | Somewhat important | 3 (6) | 8 (7) | 11 (7) |  |
|  | Quite important | 13 (26) | 30 (25) | 43 (25) |  |
|  | Very important | 11 (22) | 56 (48) | 67 (40) |  |
|  | Total | 51 | 118 | 169 |  |
|  | | | | | |
|  | Answer P-BAS-P | Answer RSCL | | Total | Cramér’s V |
|  |  | n (% within RSCL) | | |  |
|  |  | no | yes |  |  |
| Participants who indicated diarrhoea and/ or constipation on the RSCL, are expected to have a higher importance on the goal ‘improving bowel movements’. | Doesn’t apply/  not at all important | 132 (96) | 18 (58) | 150 (89) | n.c. |
|  | Somewhat important | 0 | 3 (10) | 3 (2) |  |
|  | Quite important | 2 (1) | 4 (13) | 6 (4) |  |
|  | Very important | 4 (3) | 6 (19) | 10 (6) |  |
|  | Total | 138 | 31 | 169 |  |
|  | | | | | |
|  | Answer P-BAS-P | Answer RSCL | | Total | Cramér’s V |
|  |  | n (% within RSCL) | | |  |
|  |  | no | yes |  |  |
| Participants who indicated shortness of breath on the RSCL, are expected to have a higher importance on the goal ‘reducing shortness of breath’.* | Doesn’t apply/  not at all important | 66 (81) | 12 (14) | 78 (46) | .68 |
|  | Somewhat important | 1 (1) | 2 (2) | 3 (2) |  |
|  | Quite important | 8 (10) | 17 (20) | 25 (15) |  |
|  | Very important | 7 (9) | 56 (64) | 63 (37) |  |
|  | Total | 82 | 87 | 169 |  |
|  | | | | | |
|  | Answer P-BAS-P | Answer EQ-5D | | Total | Cramér’s V |
|  |  | n (% within EQ-5D) | | |  |
|  |  | No problems | some problems/ confined to bed |  |  |
| Participants who indicated some problems or confined to bed on the EQ-5D mobility, are expected to have a higher importance for the goal ‘improving walking’.* | Doesn’t apply/  not at all important | 43 (73) | 33 (30) | 76 (45) | .41 |
|  | Somewhat important | 0 | 5 (5) | 5 (3) |  |
|  | Quite important | 8 (14) | 23 (21) | 31 (19) |  |
|  | Very important | 8 (14) | 23 (21) | 31 (19) |  |
|  | Total | 59 | 109 | 169 |  |

* To fit the assumptions of the Cramér’s V statistic, the categories somewhat and quite important were combined.

| Hypothesis | Answer P-BAS-P | Answer EQ-5D | | Total | Cramér’s V |
| --- | --- | --- | --- | --- | --- |
|  |  | n (% within EQ-5D) | | |  |
|  |  | No problems | Some problems/ unable |  |  |
| Participants who indicated some problems or unable on the EQ-5D self-care, are expected to have a higher importance for the goal ‘improving washing/dressing’.* | Doesn’t apply/  not at all important | 100 (94) | 42 (67) | 142 (84) | .37 |
|  | Somewhat important | 0 | 1 (2) | 1 (1) |  |
|  | Quite important | 2 (2) | 10 (16) | 12 (7) |  |
|  | Very important | 4 (4) | 10 (16) | 14 (8) |  |
|  | Total | 106 | 63 | 169 |  |
|  | | | | | |
|  | Answer P-BAS-P | Admission type | | Total | Cramér’s V |
|  |  | n (% within admission type) | | |  |
|  |  | No problems | Some problems/ unable |  |  |
| Participants who indicated some problems or unable on the EQ-5D usual activities, are expected to have a higher importance for the goal ‘improving hobbies’.* | Doesn’t apply/  not at all important | 42 (78) | 79 (71) | 121 (73) | .37 |
|  | Somewhat important | 1 (2) | 8 (7) | 9 (5) |  |
|  | Quite important | 3 (6) | 9 (8) | 12 (7) |  |
|  | Very important | 8 (15) | 16 (14) | 25 (15) |  |
|  | Total | 54 | 112 | 166 |  |
|  | | | | | |
|  | Answer P-BAS-P | Admission type | | Total | Cramér’s V |
|  |  | n (% within admission type) | | |  |
|  |  | Not acute or diagnostic | Acute or diagnostic |  |  |
| Participants who had an acute admission and/ or a diagnostic admission reason, are expected to have a higher importance on the goal ‘knowing what is wrong with me’.* | Doesn’t apply/  not at all important | 55 (79) | 54 (59) | 109 (68) | .20 |
|  | Somewhat important | 0 | 2 (2) | 2 (1) |  |
|  | Quite important | 4 (6) | 8 (9) | 12 (8) |  |
|  | Very important | 11 (16) | 27 (30) | 38 (24) |  |
|  | Total | 70 | 91 | 161 |  |

* To fit the assumptions of the Cramér’s V statistic, the categories somewhat and quite important were combined.
