# Supplementary material for: Adaptation of the Patient Benefit Assessment Scale for Hospitalised Older Patients: development, reliability and validity of the P-BAS picture version
Source: BMC Geriatr. 2022 Jan 11;22:43. doi: 10.1186/s12877-021-02708-7 (PMC8751090; doi:10.1186/s12877-021-02708-7)
Supplement: Supplementary file 8 — Additional file 8. Comparison achievement of goals stated in open question and P-BAS-P scores. [file 12877_2021_2708_MOESM8_ESM.docx]

**Additional file 8. Comparison achievement of goals stated in open question and P-BAS-P scores**

**Adaptation of the Patient Benefit Assessment Scale for Hospitalised Older Patients: development, reliability and validity of the P-BAS Picture version**

**Authors:**

1. Maria Johanna van der Kluit, MSc RN (Corresponding author)

University of Groningen, University Medical Center Groningen, University Center for Geriatric Medicine, Hanzeplein 1, 9700 RB Groningen, The Netherlands

[m.j.van.der.kluit@umcg.nl](mailto:m.j.van.der.kluit@umcg.nl)

+31503613921

1. Geke J. Dijkstra, PhD

University of Groningen, University Medical Center Groningen, Department of Health Sciences, Applied Health Research, Groningen, The Netherlands

NHL Stenden University of Applied Sciences, Research Group Living, Wellbeing and Care for Older People, Leeuwarden, The Netherlands

[g.j.dijkstra@umcg.nl](mailto:g.j.dijkstra@umcg.nl)

1. Sophia E. de Rooij, MD PhD

University of Groningen, University Medical Center Groningen, University Center for Geriatric Medicine, Groningen, The Netherlands

[sejaderooij@gmail.com](mailto:sejaderooij@gmail.com)

**Additional file 8. Comparison achievement of goals stated in open question and P-BAS-P scores**

Table 1. Achievement of goals and P-BAS-P answers congruent

| Goal formulated by participant in open question | Achievement | Corresponding  item P-BAS-P | | Baseline status | Follow-up status | Score |
| --- | --- | --- | --- | --- | --- | --- |
| That I’ll be a bit fitter again and be able to do my daily activities, like housekeeping and going to the store.* | somewhat | better | improvement | mediocre | satisfactory | 0 |
| That I will have more energy due to the new wires in my ICD. | not at all | energy | improvement | bad | bad | -1 |
| That I will have less shortness of breath and fatigue when I am going home soon.* | somewhat | energy | improvement | very bad | mediocre | 1 |
| That my shortness of breath and fatigue will be better.* | somewhat | energy | improvement | bad | satisfactory | 1 |
| That I will have my energy back. | quite | energy | improvement | bad | satisfactory | 1 |
| That the chest pain will resolve.* | completely | pain | improvement | bad | good | 2 |
| That I’ll get rid of the pain (heart attack). | somewhat | pain | improvement | bad | mediocre | 0 |
| That I’ll get rid of the chest pain. | completely | pain | improvement | bad | very good | 3 |
| That I will have less shortness of breath and fatigue when I am going home soon.* | somewhat | shortness of breath | improvement | bad | satisfactory | 1 |
| Curing of a pulmonary embolism so that I have more breath.* | quite | shortness of breath | improvement | bad | good | 2 |
| To be able to take a walk. | completely | walking | preservation | satisfactory | satisfactory | 0 |
| To be able to walk 5 to 6 kilometres. | not at all | walking | improvement | mediocre | mediocre | -1 |
| To be able to walk adequately again, a bit more distances. | quite | walking | improvement | very bad | good | 3 |
| Clearness about the situation. | completely | knowing what is wrong | | mediocre | satisfactory | 0 |
| That I will get rid of retained fluids again. | completely | disease | controlling | mediocre | good | 2 |
| That I will get rid of the tumour in the colon. | completely | disease | curing | mediocre | very good | 2 |
| That the leakage of my aortic valve will be resolved. | completely | disease | curing | mediocre | good | 1 |
| That my heart functions normally again. | completely | disease | curing | mediocre | good | 1 |
| That the liver disease will be restored. | completely | disease | curing | bad | very good | 3 |
| Curing of a pulmonary embolism so that I have more breath.* | quite | disease | curing | mediocre | good | 1 |
| That the cancer will be removed at once and that I don’t need chemotherapy or radiation. | quite | disease | curing | mediocre | good | 1 |
| To solve the problem in the lungs. | completely | disease | controlling | very bad | satisfactory | 3 |

Table 1 (continued). Achievement of goals and P-BAS-P answers congruent

| Goal formulated by participant in open question | Achievement | Corresponding  item P-BAS-P | | Baseline status | Follow-up status | Score |
| --- | --- | --- | --- | --- | --- | --- |
| To resolve the fungus in the mouth. | completely | disease | controlling | very bad | satisfactory | 3 |
| Recover from the complaints/injuries caused by the fall with the bicycle. | quite | disease | curing | mediocre | satisfactory | 0 |
| Staying alive for a couple of years. | completely | alive | | | | 0 |
| That my husband and I will be together for a very long time. | quite | alive | | | | 0 |
| To prolong my life for a couple of years. | completely | alive | | | | 0 |
| Growing old together with Pete, live to be 88. | completely | alive | | | | 0 |
| Remaining alive. | completely | alive | | | | 0 |
| Enjoying nature. | not at all | enjoy | improvement | mediocre | mediocre | -1 |
| That I’ll be a bit fitter again and be able to do my daily activities, like housekeeping and going to the store.* | somewhat | groceries | improvement | bad | mediocre | 0 |
| I would like to do the groceries myself. | completely | groceries | preservation | good | good | 0 |
| Keep on gardening, cycling, walking. | completely | gardening | improvement | mediocre | good | 1 |
| To be able to play table tennis. | completely | sports | preservation | good | very good | 1 |
| To be able to fish. | completely | hobbies | preservation | good | very good | 1 |
| Doing repairs in the house. | quite | hobbies | improvement | satisfactory | good | 0 |
| That I will be able to do my daily activities after the surgery. Such as handicrafts, joining diverse activities in the senior home. | completely | hobbies | preservation | satisfactory | very good | 2 |
| Going to the model car club | not at all | hobbies | improvement | mediocre | mediocre | -1 |
| To be able to exercise my hobby completely, old music/ sit down at my computer | completely | hobbies | improvement | mediocre | good | 1 |
| Set the table, cooking, reading a book (daily activities).* | completely | hobbies | improvement | very bad | good | 3 |
| I want to be able to walk in the forest again. | completely | outings | preservation | good | good | 0 |
| Visiting the grandchildren. | completely | visit | preservation | good | good | 0 |
| Going back to the senior home | completely | home | preservation | good | good | 0 |
| Set the table, cooking, reading a book (daily activities).* | completely | Independence | improvement | very bad | good | 3 |

* Some goals correspond with two P-BAS-P items

Table 2. Achievement of goals and P-BAS-P answers contrary

| Goal formulated by participant in open question | Achievement | Corresponding  item P-BAS -P | | Baseline status | Follow-up status | Score | Explanation |
| --- | --- | --- | --- | --- | --- | --- | --- |
| Better condition to be able to walk and to climb the stairs.* | somewhat | energy | improvement | satisfactory | mediocre | -2 | According to answer on open question, the goal was ‘somewhat’ reached, but according to the P-BAS-P the energy level deteriorated. |
| To be able to have a short walk through the village again. | quite | walking | improvement | mediocre | mediocre | -1 | According to answer on open question, the goal was ‘quite’ reached, but then the answer ‘mediocre’ on the P-BAS-P is a bit too negative. |
| Having clarity about my health situation. | quite | knowing what is wrong | | good | good | -1 | The clarity was already 'good' on baseline, so there was hardly room for improvement. Was the P-BAS-P baseline answer correct? |
| I would like that my new valve will function well. | completely | disease | curing | bad | bad | -1 | When a new valve functions ‘completely’, ‘bad' is not a logical answer on the P-BAS-P. |
| Getting better, that the surgery causes that it will be alright again. | quite | disease | curing | very good | good | -2 | The disease/condition was already 'very good' on baseline, so there was no room for improvement and it was questionable why the participant needed surgery. Was the P-BAS-P baseline answer correct? |
| That there will be no gallstones anymore that can cause pancreatitis. | completely | disease | curing | very good | very good | -1 | The disease/condition was already 'very good' on baseline, so there was no room for improvement and it was questionable why the participant needed surgery. Was the P-BAS-P baseline answer correct? |
| That I will get rid of my rhythm disturbance. | quite | disease | curing | good | good | -1 | The disease/condition was already ‘good' on baseline, so there was hardly room for improvement and it was questionable why the participant needed a cure. Was the P-BAS-P baseline answer correct? |
| That I’m able to sport a bit more. | not at all | sports | improvement | bad | good | 2 | According to answer on open question, the goal was ‘not at all’ reached, but according to the P-BAS-P sports is ‘good’. |

Table 2 (continued). Achievement of goals and P-BAS-P answers contrary

| Reading, watching television. | quite | hobbies | improvement | bad | bad | -1 | According to answer on open question, the goal was ‘quite’ reached, but according to the P-BAS-P, it still goes ‘bad’ with the hobbies. Is the participant meaning the same hobbies? |
| --- | --- | --- | --- | --- | --- | --- | --- |
| To be able to go back to my senior home. | completely | home | preservation | good | very bad | -4 | Answers seem to be completely contrary. The participant was at the follow-up moment in a temporary nursing home and not in her own senior home, so the P-BAS-P answer ‘very bad’ appears to be logical. The participant explained when giving the answer ‘completely’ to the open question: 'I reached the goal, however, due to a second pneumonia, I am elsewhere now.’ |

* Some goals correspond with two P-BAS-P items

Table 3. Achievement of goals and P-BAS-P answers half congruent, or dubious cases.

| Goal formulated by participant in open question | Achievement | Corresponding  item P-BAS P | | Baseline status | Follow-up status | Score | Explanation |
| --- | --- | --- | --- | --- | --- | --- | --- |
| That I will have more energy | somewhat | energy | improvement | bad | good | 2 | Improvement on P-BAS-P is larger than expected with ‘somewhat’ on the open question. |
| That my shortness of breath and fatigue will be better.* | somewhat | Shortness of breath | improvement | bad | good | 2 | Improvement on P-BAS-P is larger than expected with ‘somewhat’ on the open question. |
| Better condition to be able to walk and to climb the stairs.* | somewhat | walking | improvement | satisfactory | good | 0 | The answer 'good' given on P-BAS-P FU, does not correspond with ‘somewhat’ on the open question. |
| To be able to walk for half an hour. | somewhat | walking | improvement | mediocre | good | 1 | Improvement on P-BAS-P is larger than expected with ‘somewhat’ on the open question. Could there be recalibration, and is walking shorter than half an hour good? |

Table 3 (continued). Achievement of goals and P-BAS-P answers half congruent, or dubious cases.

| Having clarity about the cause of the pain and the shortness of breath. | somewhat | knowing what is wrong | | mediocre | good | 1 | The answer ‘somewhat' is a bit too negative for a person stating the clearness is 'good' on follow-up. |
| --- | --- | --- | --- | --- | --- | --- | --- |
| That the chest pain will resolve.* | completely | disease | curing | mediocre | satisfactory | 0 | If the chest pain is ‘completely’ resolved, one would expect the answer 'good' instead of ‘satisfactory’ on the P-BAS. |
| Removing spot on the liver. | completely | disease | controlling | satisfactory | satisfactory | 0 | ‘Satisfactory’ might be too optimistic on baseline, however, it is a subjective scale, so it could be possible. |

* Some goals correspond with two P-BAS-P items
